# Autoimmunity and immunodeficiency associated with monoallelic *LIG4* mutations via haploinsufficiency

**DOI:** 10.1016/j.jaci.2023.03.022

**Published:** 2023-03-31

**Authors:** Annaïse J. Jauch, Olivier Bignucolo, Sayuri Seki, Marie Ghraichy, Ottavia M. Delmonte, Valentin von Niederhäusern, Rebecca Higgins, Adhideb Ghosh, Masako Nishizawa, Mariko Tanaka, Adrian Baldrich, Julius Köppen, Julia R. Hirsiger, Robin Hupfer, Stephan Ehl, Anne Rensing-Ehl, Helmut Hopfer, Spasenija Savic Prince, Stephen R. Daley, Florian A. Marquardsen, Benedikt J. Meyer, Michael Tamm, Thomas D. Daikeler, Tamara Diesch, Thomas Kühne, Arthur Helbling, Caroline Berkemeier, Ingmar Heijnen, Alexander A. Navarini, Johannes Trück, Jean-Pierre de Villartay, Annette Oxenius, Christoph T. Berger, Christoph Hess, Luigi D. Notarangelo, Hiroyuki Yamamoto, Mike Recher

**Affiliations:** a Immunodeficiency Laboratory, University of Basel and University Hospital of Basel; b Swiss Institute of Bioinformatics, Basel; c AIDS Research Center, National Institute of Infectious Diseases, Tokyo; d Division of Immunology and Children’s Research Center, University Children’s Hospital Zurich, University of Zurich; e Laboratory of Clinical Immunology and Microbiology, National Institute of Allergy and Infectious Diseases, National Institutes of Health, Bethesda; f Division of Dermatology and Dermatology Laboratory, University of Basel and University Hospital of Basel; g Competence Center for Personalized Medicine, University of Zürich/Eidgenössische Technische Hochschule; h Department of Pathology, The University of Tokyo; i Translational Immunology, University of Basel and University Hospital of Basel; j Institute for Immunodeficiency, Center for Chronic Immunodeficiency, Medical Center, Faculty for Medicine, University of Freiburg; k Institute for Pathology, University Hospital Basel; l Centre for Immunology and Infection Control, School of Biomedical Sciences, Faculty of Health, Queensland University of Technology, Brisbane; m Department of Pneumology, University Hospital Basel; n Department of Rheumatology, University Hospital Basel; o University Center for Immunology, University Hospital Basel; p Division of Pediatric Oncology/Hematology, University Children’s Hospital Basel; q Division of Allergology and clinical Immunology, Department of Pneumology and Allergology, Inselspital, Bern University Hospital, University of Bern; r Division Medical Immunology, Laboratory Medicine, University of Basel and University Hospital of Basel; s Laboratory of Genome Dynamics in the Immune System, Institut National de la Santé et de la Recherche Médicale Unité Mixte de Recherché 1163, Université Paris Descartes Sorbonne Paris Cité, Institut Imagine; t Institute of Microbiology, Eidgenössische Technische Hochschule, Zurich; u Immunobiology Laboratory, Department of Biomedicine, University of Basel and University Hospital of Basel; v Cambridge Institute of Therapeutic Immunology and Infectious Disease, Department of Medicine, University of Cambridge.

**Keywords:** DNA ligase 4, DNA damage–autoimmunity, haploinsufficiency, autosomal dominant, inborn errors of immunity, immunodeficiency, primary immunodeficiency

## Abstract

**Background:**

Biallelic mutations in *LIG4* encoding DNA-ligase 4 cause a rare immunodeficiency syndrome manifesting as infant-onset life-threatening and/or opportunistic infections, skeletal malformations, radiosensitivity and neoplasia. LIG4 is pivotal during DNA repair and during V(D)J recombination as it performs the final DNA-break sealing step.

**Objectives:**

This study explored whether monoallelic *LIG4* missense mutations may underlie immunodeficiency and autoimmunity with autosomal dominant inheritance.

**Methods:**

Extensive flow-cytometric immune-phenotyping was performed. Rare variants of immune system genes were analyzed by whole exome sequencing. DNA repair functionality and T-cell–intrinsic DNA damage tolerance was tested with an ensemble of *in vitro* and *in silico* tools. Antigen-receptor diversity and autoimmune features were characterized by high-throughput sequencing and autoantibody arrays. Reconstitution of wild-type versus mutant *LIG4* were performed in *LIG4* knockout Jurkat T cells, and DNA damage tolerance was subsequently assessed.

**Results:**

A novel heterozygous LIG4 loss-of-function mutation (p.R580Q), associated with a dominantly inherited familial immune-dysregulation consisting of autoimmune cytopenias, and in the index patient with lymphoproliferation, agammaglobulinemia, and adaptive immune cell infiltration into nonlymphoid organs. Immunophenotyping revealed reduced naive CD4^+^ T cells and low TCR-Vα7.2^+^ T cells, while T-/B-cell receptor repertoires showed only mild alterations. Cohort screening identified 2 other nonrelated patients with the monoallelic *LIG4* mutation p.A842D recapitulating clinical and immune-phenotypic dysregulations observed in the index family and displaying T-cell–intrinsic DNA damage intolerance. Reconstitution experiments and molecular dynamics simulations categorize both missense mutations as loss-of-function and haploinsufficient.

**Conclusions:**

This study provides evidence that certain monoallelic *LIG4* mutations may cause human immune dysregulation via haploinsufficiency.

The 3 mammalian DNA ligases (LIG1, LIG3, LIG4) are pivotal for genomic recombination, replication, and repair.^1^ LIG4 is essential for resolving DNA double-strand breaks (DSBs)—the most noxious DNA lesions.^2^ DSB mending engages the ubiquitous nonhomologous end-joining (NHEJ) repair pathway, which uses LIG4 for the last step of DNA religation.^2^

NHEJ is preferentially used after genotoxic assaults such as ionizing radiation (IR) as well as physiologically during V(D)J recombination, a crucial step in the T- and B-cell receptor generation (TCR and BCR, respectively).^3^ V(D)J recombination is mandatory for the development of adaptive immunity, as the variability and, consequently, the antigen recognition is ensured by the semistochastic recombination of the variable (V), diversity (D), and joining (J) gene segments encoding the variable domains of both TCRs and BCRs.^3^ A well-regulated DNA-damage response is therefore imperative for immune homeostasis and to guarantee immunocompetence and immune tolerance.

Although the first LIG4-deficient patient was characterized 33 years ago, only 120 patients with either homozygous or compound heterozygous mutated *LIG4* have been published to date (reviewed in Table I).^4–44^ LIG4 haploinsufficiency caused by monoallelic *LIG4* mutations has not been reported in human patients, whereas murine data suggest that a single functional *LIG4* allele may not be sufficient to protect from malignancy and may reduce survival.^45–47^ Here we identified 2 novel monoallelic *LIG4* missense variants associated with impaired tolerance to DNA damage in primary T cells and combined immunodeficiency, in 4 patients from 3 nonrelated families.

## METHODS

### Ethics approval and human subjects

Following informed consent, the patients and family members were included into a prospective cohort that was approved by the Ethics Committee of the Northwestern and central Switzerland (EKNZ 2015–187), complying with all national and international ethical regulations. Blood samples from healthy donors were obtained after informed consent from the Blood Donor Center, University Hospital Basel.

### Genetic analysis

Genomic DNA was isolated from cultured T-cell blasts or PBMCs using the QIAamp DNA Blood Mini Kit (Qiagen, Venlo, The Netherlands). Whole exome sequencing was performed as described earlier.^48,49^

The *LIG4* variant was confirmed by Sanger sequencing of PCR amplification products of cDNA derived from PBMCs. After running the amplicon on a 1.5% agarose gel, DNAwas extracted with QIAquick Gel Extraction Kit (Qiagen). The purified PCR products were then bidirectionally sequenced by Microsynth (Balgach, Switzerland).

### Cell isolation and immunophenotyping

Patient- and healthy control–derived PBMCs were isolated from whole blood, via Ficoll density gradient separation using Lymphoprep (density 1.077 g/mL; Axonlab, Baden, Switzerland).

Cells were stained in PBS containing 2.5% human albumin serum, NaH_3_ 0.01%, HEPES 25 mmol/L, Fc block (#426101; BioLegend, San Diego, Calif) for 30 minutes at 4°C. Chemokine receptor staining was performed at 37°C for 20 minutes. All primary/secondary antibody conjugates are listed in the Methods section in this article’s Online Repository at www.jacionline.org. Cell viability was assessed using Live/Dead Fixable NIR (#L34975, Invitrogen, Thermo Fisher Scientific, Waltham, Mass). Data analysis was performed using FlowJo software (version 10.5.2; TreeStar, BD, Franklin Lakes, NJ).

Additional methods are reported in the Methods section in this article’s Online Repository.

## RESULTS

### Dominantly inherited immune-dysregulation

**FIG 1.**
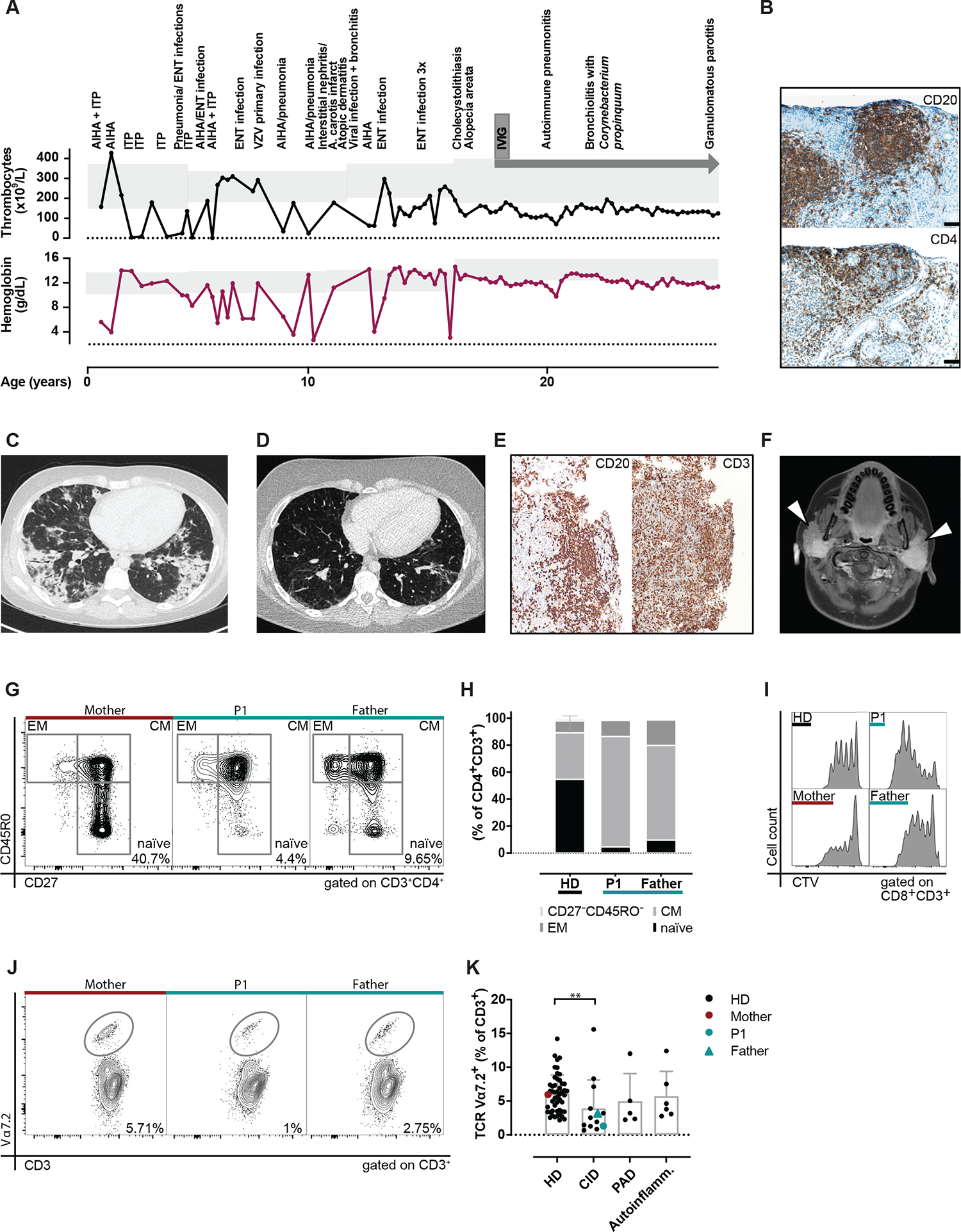
MultipleautoimmunemanifestationsandreductionofnaiveTcellsintheperipheralbloodofP1andher father. **(A)** Clinical manifestations in the index patient P1, thrombocyte counts, and hemoglobin levels; *gray* background depicts reference range. **(B)** P1’s kidney biopsy during interstitial nephritis. Immunohistochemistry staining with anti-CD20 and anti-CD4. **(C)** Pulmonary tissue gated computer tomography scan of P1 during the pneumonitis episode and **(D)** after steroid treatment. **(E)** Lung biopsy specimens during the pneumonitis episode and stained with anti-CD20 and anti-CD3. **(F)** Cranial magnetic resonance imaging, showing parotid gland swelling (*white arrowheads*). **(G)** Peripheral blood T-cell subsets with naive (CD27^+^CD45RO^−^), effector memory (*EM*; CD27^−^CD45RO^+^) and central memory (*CM*; CD27^+^CD45RO^+^), and **(H)** quantification. **(I)** Cell Trace violet (*CTV*) dilution after 5 days of *in vitro* stimulation. **(J)** Enumeration of T cells bearing the TCR Vα7.2 segment by flow-cytometry. The number indicates the frequency within the CD3^+^ T-cell population. **(K)** Comparison of the TCR Vα7.2^+^ T-cell frequency in P1 and her father with patients affected by combined immunodeficiency (*CID*), primary antibody deficiency (*PAD*), autoinflammation (*Autoinflamm*) or to HDs. **(K)** Non-parametric Kruskal-Wallis test with Dunn’s correction. **P < .01. *ENT*, Ears, nose, and throat; *ITP*, immune thrombocytopenia; *IVIG*, intravenous immunoglobulin; *VZV*, varicella-zoster virus.

Patient 1 (P1), presented at the age of 2 years with autoimmune hemolytic anemia and immune thrombocytopenia (Fig 1, A). During the disease course, P1 developed lymphoproliferation (splenomegaly and lymphadenopathy) and multiple infections including opportunistic pathogens (Fig 1, A). At the age of 11 years, P1 developed biopsy-proven interstitial nephritis with polyclonal T- and B-cell infiltrations (Fig 1, B). At the transition into the adult immunology service, being under immune suppression with mycophenolate, agammaglobulinemia was noted. Immunoglobulin replacement therapy was started at this time. Despite normalized serum IgG levels, P1 developed life-threatening noninfectious pneumonitis, again characterized by polyclonal lymphocyte infiltration (Fig 1, C–E). Lastly, sterile granulomatous parotitis was diagnosed (Fig 1, F). Her father and 2 paternal uncles experienced several adult-onset immune thrombocytopenia episodes that responded to systemic steroids.

A detailed immunological evaluation was performed in P1 and her father (P2). The father had mildly reduced lymphocytes (1.02 × 10^9^/L) and thrombocytes (114 × 10^9^/L), in the absence of immune-modulating treatment (Table E1 in this article’s Online Repository at www.jacionline.org). Analysis of PBMCs revealed a reduced frequency of naive CD27^+^CD45RO^−^ T cells in both patients (Fig 1, G and H). T-cell proliferation on mitogen stimulation was enhanced(Fig 1, I). Peripheral blood-derived CD4^+^ regulatory T cells (CD25^hi^CD127^low^) were reduced in frequency in both P1 and her father compared to healthy donors (HDs) (Fig E1, A in this article’s Online Repository at www.jacionline.org). Those regulatory T cells displayed an activated and proinflammatory phenotype (Fig E1, B). CD4^+^ T cells also displayed a phenotype skewed toward T_H_1 (Fig E1, C). Autoreactivity of B cells was investigated by probing the father’s serum immunoglobulins against different self-antigens on a protein microarray and compared with sex-matched controls. Four of the tested IgG autoantibody specificities were found to be elevated in the serum of the father (Fig E1, D and F), including augmented IgG directed against genomic DNA (Fig E1, E). Endogenous IgG of P1 could not be tested due to the agammaglobulinemia and the immunoglobulin substitution. Low T cells bearing the TCR Vα7.2^+^ were noted in both (Fig 1, J), similarly to what was found in some other patients diagnosed with combined immunodeficiency in our cohort (Fig 1, K).

Because low TCRVα7.2^+^ T cells have been reported as a hallmark observed in patients with V(D)J recombination defects,^50,51^ we performed TCR and BCR high-throughput sequencing.

### Preserved TCR/BCR repertoires

**FIG 2.**
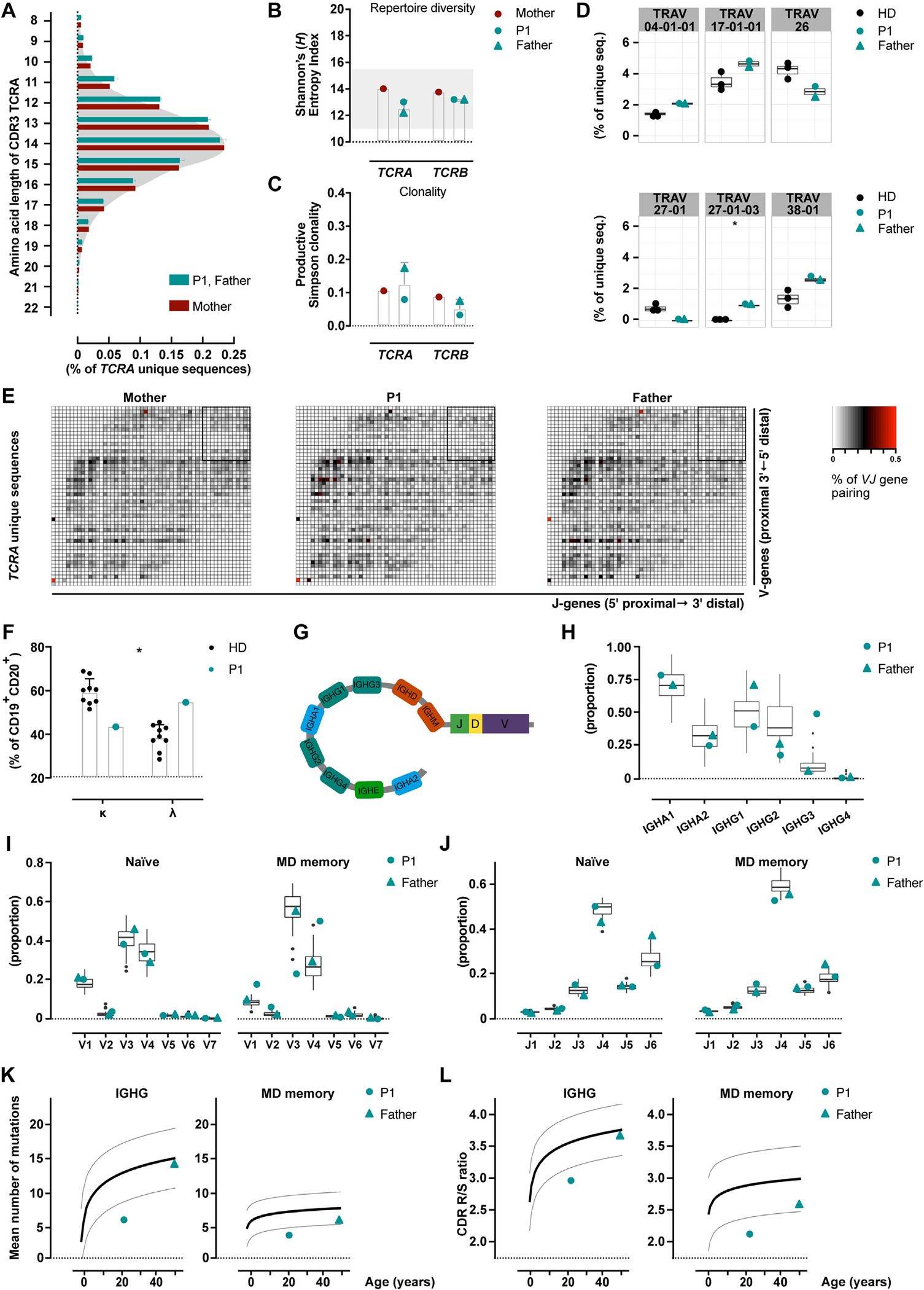
Preserved BCR and TCR repertoires. **(A)** High-throughput sequencing of the TCR loci. Complementarity-determining region 3 (*CDR3*) length distribution. **(B)** Shannon’s (*H*) entropy index; *gray shadow* for HD values.^52^ **(C)** Simpson clonality index. **(D)** Individual V gene segment usage. **(E)** Heatmaps displaying VJ gene pairing; box indicates most distal gene pairing. **(F)** Surface expression of the BCR light chains. **(G)** IGH locus cartoon for the constant region (adapted from Bashford-Rogers et al^53^). IGH high-throughput RNA-sequencing for the determination of B-cell maturation status and constant region gene usage. **(H)** IgA and IgG subclass utilization. Box-plot indicates age-matched HDs values. **(I)** V family and **(J)** J gene segment usage. Box-plot indicates values of age-matched HDs. **(K)** Average of somatic hypermutations. The *black line* indicates the model fitting the somatic hypermutations increase by age; *gray lines* indicate the 95% CI. **(L)** Antigen selection was quantified by the computation of the mean R/S ratio. The *black line* indicates the model fitting and the R/S increase by age; *gray lines* indicate the 95% CI. **(D)** Differential expression analysis empirical Bayes method. **(F)** Mann-Whitney test with *post hoc* correction; the HDs’ SD was added to the value of P1. *TRAV*, T cell receptor alpha variable gene.

The most common TCR loci were sequenced, using DNA derived from peripheral blood T cells from P1 and her parents. The distribution of the most variable region of the TCR, the complementarity-determining region 3 lengths in the T-cell receptor α-chain (TCRA) (Fig 2, A)^52,53^ and β-chain sequences (Fig E2, A in this article’s Online Repository at www.jacionline.org) were comparable in P1 and her parents. To account for the entire repertoire diversity and clonality, the Shannon’s (*H*) entropy^54^ and Simpson’s clonality^55^ indices were computed and found to be normal (Fig 2, B and C, respectively).

We focused on the individual TCRA V gene segment usage, because this locus can adopt a directional multistage recombination, which is halted only on positive thymocyte selection.^56^ We found only the V-gene segment 27–01-03 to be significantly overrepresented in the 2 patients compared with in HDs (Fig 2, D).

To investigate the pairing of TCRA V with J gene segments, heatmaps were computed. The pairing was overall maintained, in total (Fig E2, B) as well as in unique TCRA sequences (Fig 2, E), including distal gene segment pairing (Fig 2, E and Fig E2, B).

The autoimmune disposition in P1 and her father could reflect differences in B-cell subsets and/or BCR repertoire, thus peripheral blood B cells were immunophenotyped and RNA-derived immunoglobulin heavy chain (IGH) repertoires were sequenced using isotype-resolved barcode-based adaptive immune receptor repertoire-sequencing technology.

P1 displayed an inverted BCR light chain (κ vs λ) expression on B cells compared to HDs (Fig 2,F). Both patients had an increased percentage of CD21^low^ B cells (Table E1). The vast majority of P1’s B cells included unmutated naive and memory IgM/IgD (MD memory) transcripts (Fig E2, C). Furthermore, the constant region segment utilization was investigated (Fig 2, G). In P1 IgG (*IGHG*) and IgA (*IGHA*) transcripts were barely detectable (Fig E2, C). Both patients displayed a tendency for a reduced *IGHG2* subclass frequency (Fig 2, H). In addition, P1’s B-cell transcripts showed a skewing toward the utilization of the *IGHG3* subclass (Fig 2, H).

P1’s MD memory B cells had an increased usage of the V_H_4 gene family at the expense of V_H_3 (Fig 2, I). In both patients, the memory MD B-cell transcripts harbored less abundantly the J_H_4 gene segment (Fig 2, J).

Affinity maturation was analyzed via the quantification of somatic hypermutations detected in memory B-cell transcripts, being below the normal range for P1 and marginally low in the paternal memory MD compartment (Fig 2, K). An increased ratio of replacement mutations (R) compared to silent mutations (S) (R/S ratio) in the complementarity-determining regions may point at antigen selection.^57,58^ P1’s *IGHG* and memory MD B-cell transcripts showed a decreased R/S ratio compared to HDs (Fig 2, L), while in the father’s B cells, the R/S ratio was only marginally low in MD memory B cells (Fig 2, L).

### Novel heterozygous *LIG4* missense variant

**FIG 3.**
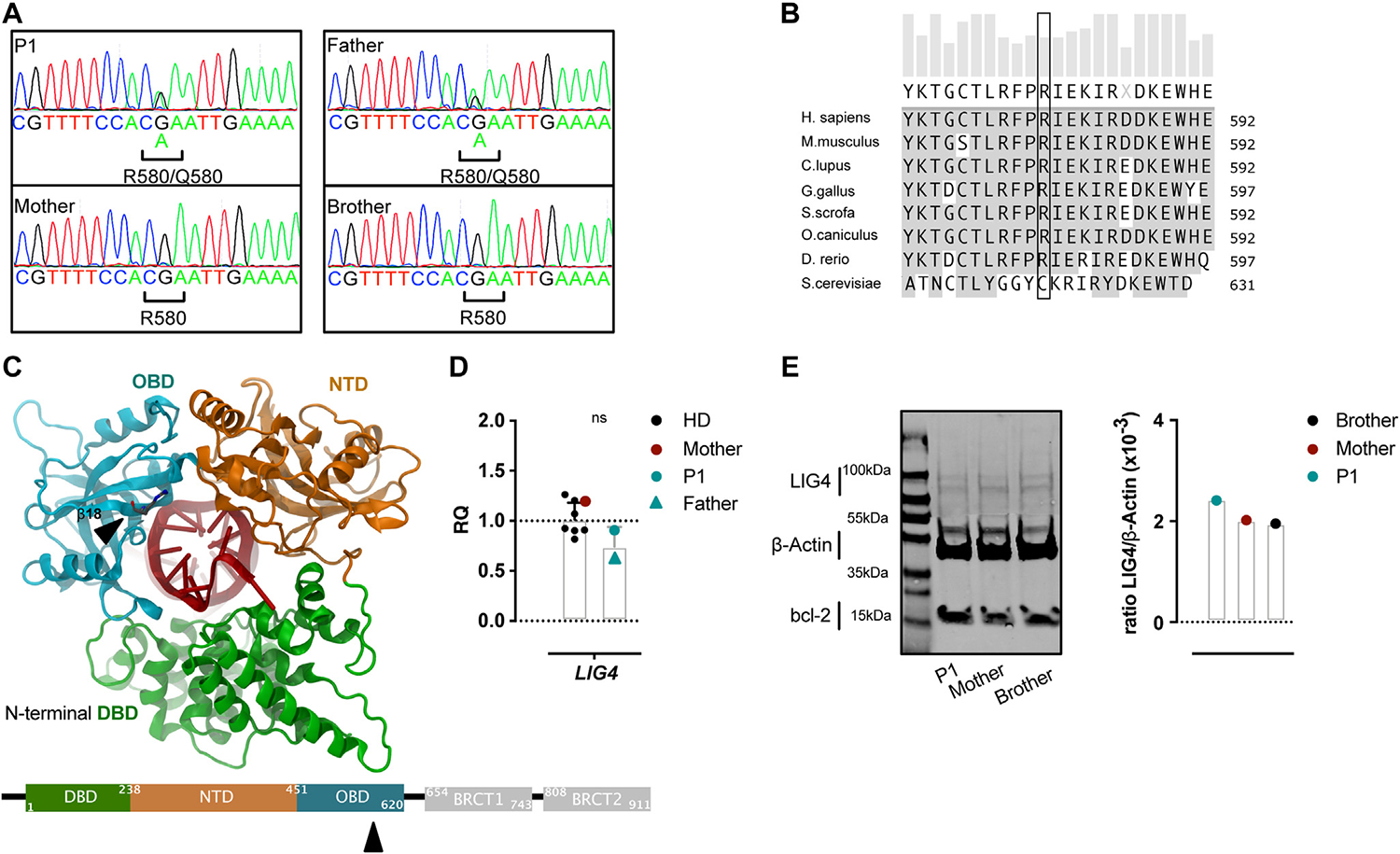
Novel missense variant within the catalytic core of LIG4. **(A)** Sanger sequencing of c.A1739G in bulk T-cell–derived DNA, the resulting amino acid change at p.R580Q is indicated. **(B)** Multiple LIG4 protein sequence alignment; p.580 position is highlighted. **(C)** Molecular representation in ribbons of the human LIG4 catalytic core bound to a DNA duplex. The WT Arg580 is shown as stick (*arrow*). The corresponding β sheet 18 is indicated. The mutated amino acid resides in the catalytic oligonucleotide/oligosaccharide-fold domain (*OBD; blue*). Numbers indicate the amino acid position in NP_001091738. DNA binding domain (*DBD*) in *green*; nucleotidyltransferase (*NTD*) in *orange*. **(D)** Qualitative PCR was used to measure *LIG4* mRNA levels in PBMCs of the 2 patients and healthy controls including the mother. The relative quantity (*RQ*) was normalized to multiple housekeeping genes and to the mean of the HDs. **(E)** The LIG4 protein levels were quantified by separating PHA T-cell blast-cell lysates by SDS-PAGE electrophoresis and probed with rabbit-anti LIG4. *Right side* normalization of LIG4 protein levels to β-actin levels. **(D)** Nonparametric Mann-Whitney rank test. *ns*, Not significant.

We next investigated PBMC-derived DNA of P1, her parents, and the clinically healthy brother using whole exome sequencing, followed by custom-designed primary immunodeficiency gene panel filtering. In both diseased individuals we detected a c.G1739A heterozygous missense variant in *LIG4* (Table E2 in this article’s Online Repository at www.jacionline.org). Sanger sequencing confirmed heterozygosity. Both the healthy mother and brother did not carry the *LIG4* variant (Fig 3, A). The c.G1739A variant causes replacement of an arginine at position 580 by a glutamine (p.R580Q). The Arg580 is highly conserved across various vertebrates (Fig 3, B), locates within the oligonucleotide/oligosaccharide-binding domain, and is crucial for complete LIG4 encirclement of the DNA during NHEJ^59^ (Fig 3, C). The variant is predicted to have functional impact on the LIG4 protein (Combined Annotation Dependent Depletion [CADD] score 33,^60^ Polymorphism Phenotyping v2 [PolyPhen-2]^61^ score 1, and Sorting Intolerant from Tolerant [SIFT]^62^ score 0) (Table E2). This *LIG4* variant has so far not been described in the literature (Table I). *LIG4* mRNAwas somewhat low in the father when compared to HDs but was normal in P1 (Fig 3, D). Immunoblots from T-cell blast-derived protein revealed conserved LIG4 protein levels in P1 (Fig 3, E).

In addition, a novel homozygous missense variant in *FAS* (c.G383A, p.R128K) (Table E2) was detected in the father. Both children, P1 and her healthy brother, were heterozygous carriers for this *FAS* variant. Based on unobtrusive FAS-related serum biomarkers, normal FAS-related apoptosis studies in T-cell blasts of P1 and the fact that the healthy brother carried the same heterozygous *FAS* variant, we excluded the rare *FAS* variant to drive the disease in P1 and her father (Fig E3, A–E in this article’s Online Repository at www.jacionline.org). Furthermore, a structure analysis predicted the extracellular R128K FAS mutation to be functionally conservative (Fig E3, E)

### The R580Q variant reduces DSB ligation and DNA binding

**FIG 4.**
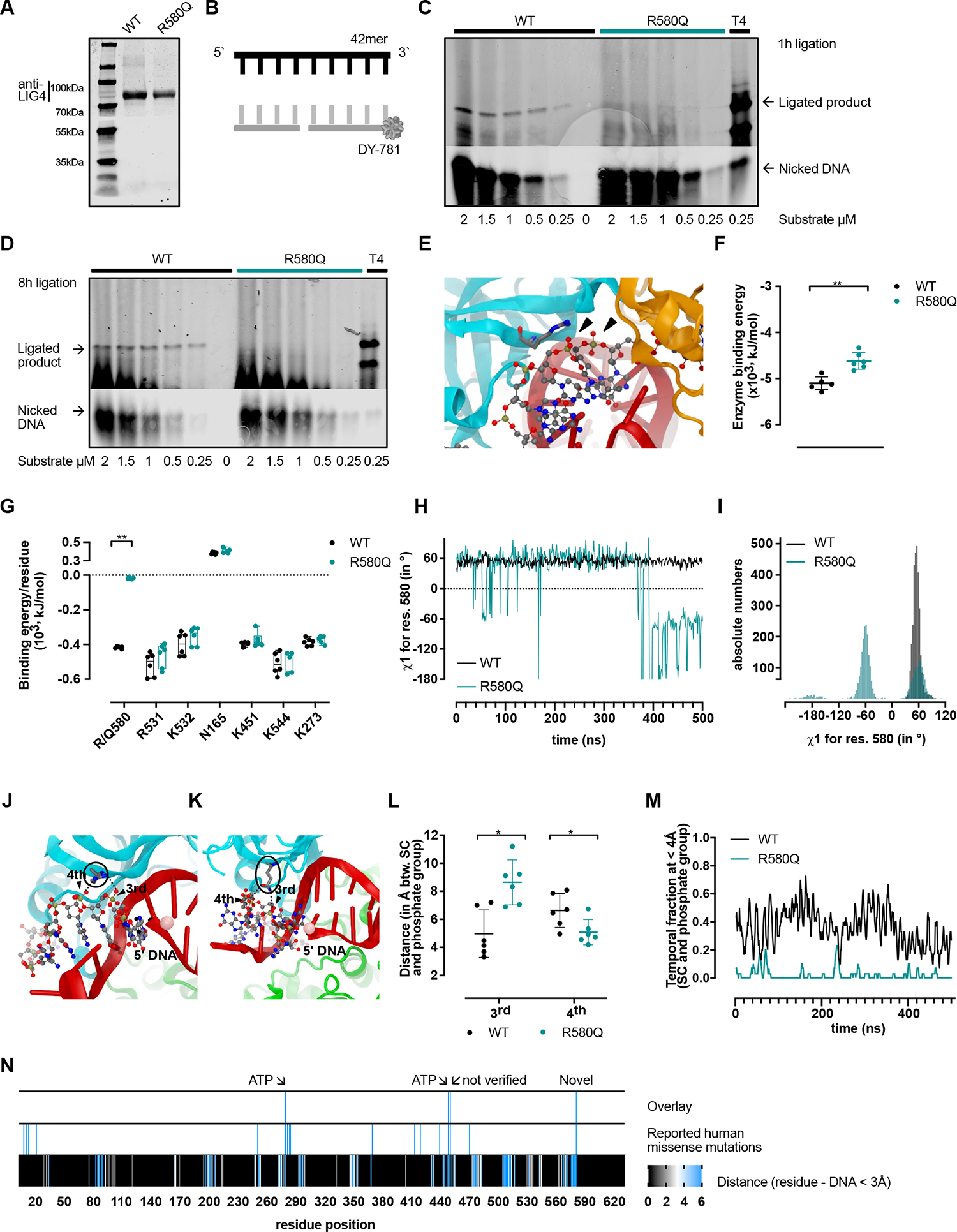
LIG4 R580Q reduces DNA-ligation activity and weakens DNA binding. **(A)** Normalization of recombinant WT or R580Q LIG4 proteins. **(B)** 42mer nicked DNA duplex. Multiple turnover ligations for WT versus R580Q LIG4 with **(C)** increasing unadenylated 42mer concentrations and **(D)** time. Product separation on a Tris/Borate/EDTA (TBE)-urea polyacrylamide gel. **(E)** Molecular OBD representation, the Arg580 represented as stick (*arrows*: nearby DNA-backbone phosphorous atoms). **(F)** Computed LIG4 binding energy (*BE*) between the WT versus R580Q LIG4 and adenylated-DNA complex. Twelve independent trajectories, each >500 nanoseconds. **(G)** Residues with BE difference >20 kJ/mol between WT and R580Q. **(H)** Dihedral χ1 angle time series and **(I)** distribution focused on residue 580. **(J)** WT LIG4 and **(K)** R580Q LIG4 (stick) with the adenylated nicked-DNA as ball and stick; third and fourth phosphate group of DNA backbone (*arrows*). **(L)** Minimal distance between the residue side chain (*SC*) and DNA backbone phosphate groups. The phosphate group numbering is indicated. **(M)** Temporal fraction, during which residue 580 SC and the DNA backbone phosphate were <4 Å. **(N)** *Bottom*: Identification of likely DNA-interacting residues (distance to DNA <3 Å). *Middle*: Human *LIG4* missense mutations (Table I). *Top*: Missense mutations with potential DNA binding. Mann-Whitney testing **(F)** with multiple comparison correction **(L); (G)** 2-way ANOVA with Šídàk correction.

The clinical phenotype of the *LIG4* variant carriers pointed to a protein loss of function associated with the R580Q variant. We performed substrate ligation assays comparing the enzymatic activity of recombinant wild-type (WT) versus mutant (R580Q) LIG4 protein (Fig 4, A). As substrate, a 42-bp nicked oligonucleotide duplex (42mer) with attached fluorescent dye was used (Fig 4, B). Applying increasing substrate concentration (Fig 4, C) and reaction duration (Fig 4, D), we observed reduced amounts of ligated products in the R580Q LIG4 presence as compared to WT.

Reduced biochemical ligation activity of the mutant R580Q LIG4 prompted us to study the LIG4-DNA interaction at the structural level. We performed molecular dynamics simulations, an approach allowing to efficiently interpret the effect of mutations on protein function.^49,63,64^ The simulations focused on the catalytic domain of LIG4 in closed conformation with a nicked adenylated-DNA substrate (PDB 6BKG). Twelve independent unbiased trajectories of >500 nanoseconds, 6 for the WT and 6 for the R580Q mutant were computed. The Arg580 interacts with the broken 5’ AMP-carrying DNA strand, with its guanidium moiety at a salt bridge distance from 2 phosphate groups (Fig 4, E) likely stabilizing the protein-DNA complex. Using the Molecular Mechanics Poisson–BoltzmannSurfaceAreaapproach,^65–67^ we calculated the free binding energy between the WT and R580Q LIG4 to the DNA. We found that the binding energy was lower in the case of the R584Q ligand (Fig 4, F and G, and Fig E4, A and B in this article’s Online Repository at www.jacionline.org). The weakened R580Q LIG4-DNA binding could not be compensated by any of the 632 neighboring residues (Fig E4, B). Thus, the residue 580 accounted alone for the largest binding energy reduction.

Next, we focused the conformational analysis on the interactions of the residue with the DNA backbone and on their torsion angles. The dihedral χ1 angle indicates the orientation of the sidechain with respect to the protein mainchain. The WT Arg580 experienced negligible oscillations in all trajectories, while the mutant Gln580 displayed greater dihedral χ1 angle fluctuations including a bimodal χ1 angle orientation (Fig 4, H and I). This suggested that Gln580 was still sampling new conformations after 500 nanoseconds. The fluctuations of Gln580 affected the secondary structure, causing a strong increase of the backbone torsion angles ϕ and ψ dynamics (Fig E4, C–F). Quantification of either the salt bridges and hydrogen bonds formed between WT Arg580 and, respectively, mutant Gln580 and the DNA (Fig 4, J–L) disclosed a higher abundance of salt bridges being formed for the WT (Fig 4, M, Fig E4, G) and significantly outnumbering the weaker hydrogen bonds for the mutant R580Q with the DNA (Fig 4, M, Fig E4, H, and Video E1 in this article’s Online Repository at www.jacionline.org).

Several mutations affecting the LIG4 catalytic domain have been reported. We wondered whether any of the previously reported mutations (Table I) would be related to DNA binding, similarly to the one characterized here. The locations of all human missense mutations affecting the LIG4 catalytic domain were compared to those of the trajectories in which the distance between enzyme and DNA was ≤3 Å Three residues other than the Arg580 were identified: p.278, p.447, and p.449 (Fig 4, N). The positions p.278 and p.449 are well-described ATP-binding residues and a biochemical characterization for the p.447 mutation was not found in the literature. Consequently, the description here of the mutation at p.580 is to our knowledge the first with experimental evidence for reduced LIG4-DNA binding.

### Dysregulated DSB repair response in heterozygous *LIG4* mutated primary T cells

To experimentally address LIG4 functionality in the context of a heterozygous missense variant, we characterized the DSB response in T cells of the patients *in vitro*.

**FIG 5.**
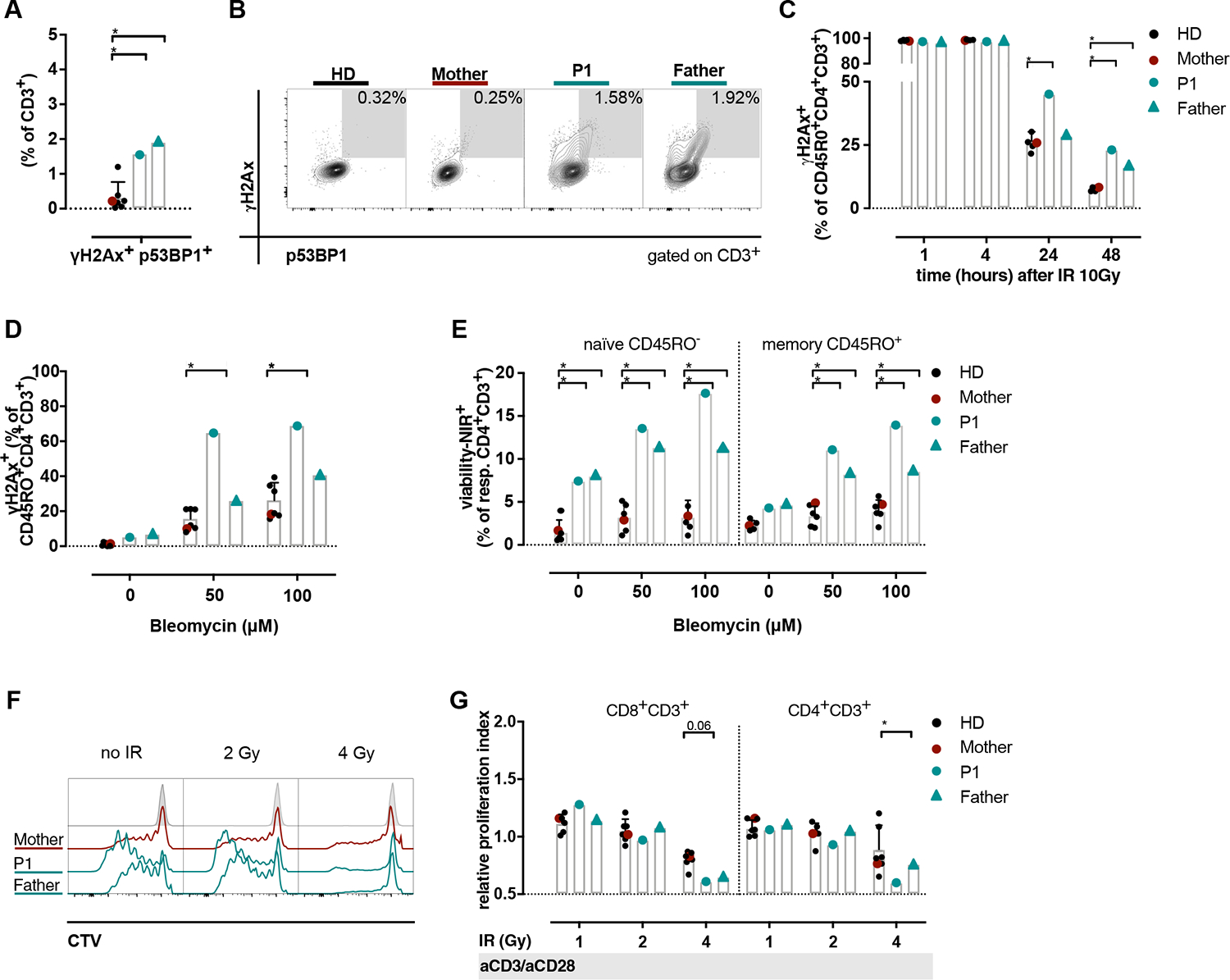
Augmented DNA-damage susceptibility *in vitro*. T cells derived from PBMCs were cultured for 2 days without stimulation. The phosphorylation of H2Ax (γH2Ax) and 53BP1 (p53BP1) were assessed by flow cytometry. **(A)** Quantification (mean of triplicates) and **(B)** representative flow cytometric plots of the γH2Ax^+^p53BP1^+^ population in bulk CD3^+^ T cells. **(C)** Kinetics of γH2Ax in CD45R0^+^CD4^+^ helper T cells after 10 Gy irradiation (*IR*). **(D)** Analysis of the nuclear γH2Ax^+^ fraction in memory CD45R0^+^ CD4^+^ T cells after *in vitro* treatment of PBMCs with bleomycin sulfate for 24 hours at indicated concentrations. **(E)** Cell death after 24 hours *in vitro* bleomycin sulfate exposure of CD4^+^ T cells (naive CD45R0^−^ and memory CD45R0^+^). **(F)** T-cell proliferation after IR. T cells were labeled with CTV, followed by IR and stimulation for 5 days *in vitro* with anti-CD3/anti-CD28 (*aCD3/aCD28*). *Gray-shaded* population indicates the maternal nonstimulated condition of T cells. **(G)** The relative proliferation index was computed for CD4^+^ and CD8^+^ T cells after different IR intensities; stimulation of cells as in **(F). (A)** Kruskal-Wallis test; **(C, D, E, G)** 2-way ANOVA with Šídàk correction. Single points represent mean values of duplicates or triplicates for the patients.

After 2 days of *in vitro* culture, we observed spontaneously increased phosphorylation of 2 important DNA damage-associated proteins H2Ax (γH2Ax) and 53BP1 (p53BP1)^68,69^ in T cells of both *LIG4* variant carriers (Fig 5, A and B). Next, we measured nuclear γH2Ax kinetics after DSB induction via IR. Memory CD45R0^+^CD4^+^ T cells of both patients displayed higher gH2Ax^+^ levels beyond 48 hours after IR compared to cells from HDs (Fig 5, C). The father’s memory CD45R0^+^CD4^+^ T cells showed a trend and P1’s memory CD4^+^ T cells a distinctly augmented proportion of H2Ax phosphorylation after *in vitro* treatment of PBMCs with the DSB inducing drug bleomycin sulfate^70^ (Fig 5, D). This was paralleled by reduced cell viability after *in vitro* bleomycin sulfate exposure in naive (CD45R0^−^) and memory (CD45R0^+^) CD4^+^ T cells of both patients as compared to cells of HDs (Fig 5, E).

T-cell proliferation capacity after IR plus mitogen stimulation, was studied by labeling peripheral blood–derived T cells with CellTrace violet (Thermo Fisher Scientific). Proliferation was quantified by assessing the CellTrace violet dye dilution. With rising IR doses, we observed a trend for a decreased relative proliferation index in both CD4^+^ and CD8^+^ T cells of the 2 *LIG4* variant carriers compared to healthy T cells (Fig 5, F and G).

### The monoallelic LIG4 mutation p.A842D recapitulates impaired T-cell intrinsic DNA damage response and is linked with combined immunodeficiency

**FIG 6.**
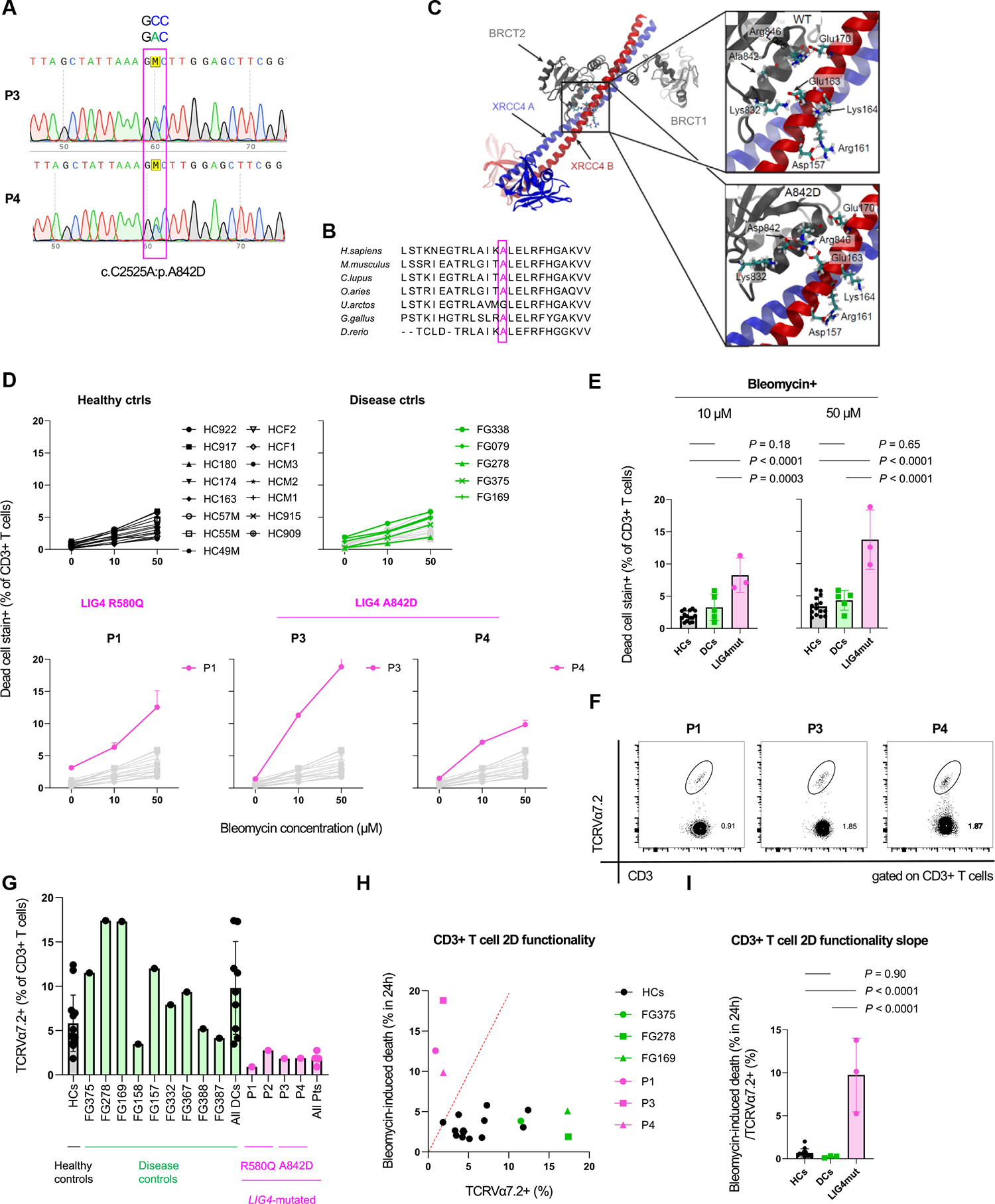
A novel LIG4 A842D mutation substantiates linkage of monoallelic *LIG4* mutations with DNA damage-induced T-cell death and immunodeficiency. **(A)** Sanger sequencing chromatogram of heterozygous LIG4 A842D mutation in P3 and P4. **(B)** Cross-species alignment of A842-proximal LIG4 residues. **(C)** LIG4-XRCC4 molecular complex highlighting residue 846-proximal area of BRCT2. Structural domains shown in *black* (BRCT1/BRCT2), *blue* (XRCC4-A), and *red* (XRCC4-B). Simulation snapshots in *boxes* for WT (*top*) and A842D (*bottom*) LIG4. Salt bridges shown as *dashed lines* when distances were mostly <5 Å during simulation. **(D)** Dead cell stain-positive frequencies (mean ± SD) in T cells following 24-hour bleomycin exposure in blood donors (n = 15, *black*); disease-controls (*DCs; green*); and P1 (R580Q), P3, and P4 (A842D). **(E)** *Post hoc* comparisons of 1-way ANOVA for bleomycin-treated groups. Representative data shown as mean of pooled triplicate/quadruplicate (P1), duplicate/triplicate (P3), or triplicate/quadruplicate (P4). **(F)** Flow-cytometric plots of TCRVα7.2^+^ T cells. **(G)** TCR Vα7.2^+^ T-cell frequencies of healthy controls (HCs; *gray*), DCs (*green*), and in patients with LIG4-mutation (*LIG4mut; pink*). **(H)** Two-dimensional plot of *ex vivo* TCRVα7.2^+^ versus *in vitro* 24-hour 50 μmol/L bleomycin-induced T-cell death. An empirical slope of 2 is appended. **(I)** One-way ANOVA of T-cell-functionality slope defined as (24-hour bleomycin-induced dead frequencies)/(TCRVα7.2-positive frequencies).

In our cohort of patients with immunodeficiency/immune dysregulation, we identified 2 additional unrelated patients (P3 and P4) carrying another functionally, so far unstudied, monoallelic *LIG4* mutation encoding p.A842D (Fig 6, A and Table E2). Rare variants in other inborn error of immunity–related genes filtered by whole exome sequencing in P3 and P4 were listed as benign or variant of unknown significance on gnomAD/ClinVar and did not align with reported clinical features or zygosity reported by the International Union of Immunologic Societies.^71^ Both were adult patients with hypogammaglobulinemia, both sharing reduced naive CD4^+^ T cells with the LIG4 p.R580Q mutation carriers of the index family (Table E1).

The alanine at position 842 is being conserved across species (Fig 6, B) within the BRCA1 C-terminal domain 2 (BRCT2) of LIG4 interacting with its cofactor XRCC4 (Fig 6, C). The distance of the proximal XRCC4 residues (Gln159, Glu163, and Val166) and LIG4 is exceeding 8 Å in a reported 2.4-Å resolution model centered around theLIG4 BRCT segment-XRCC4 interaction (PDB 3II6), implying an indirect influence of the A842D substitution on molecular interaction.^72^ We conducted 500-nanosecond-long independent unbiased MD trajectories: 4 of the WT and 4 of the A842D variant. The analyses focused on residues located within a range of 15 Å of the Cα atom of residue 842 (Fig 6, C and Fig E5 in this article’s Online Repository at www.jacionline.org). Results delineated potential alteration of a network of salt bridges involving multiple residues of XRCC4 and BRCT. A domino-effect of the A842D mutation was predicted to skew 4 pairs of acidic and basic residues located in BRCT2 and XRCC4 (Fig E5). These changes are predicted to shift binding along the XRCC4 helices (see legend of Fig E5 for detailed description). The effect of the A842D mutation was conceptually analogous to a XRCC4 R161Q mutation causing reduced DNA repair.^73^

We next readdressed immune cell-intrinsic consequences of both R580Q and A842D mutations in heterozygous state in primary T cells. Bleomycin treatment of PBMCs derived from A842D-mutated P3 and P4 resulted in significantly elevated CD3^+^ T-cell death equivalent to reanalyzed R580Q-mutated P1 (Fig 6, D and E). TCRVα7.2^+^ frequencies in T cells (Table E1 and Fig 6, F) were low similar to P1 (Fig 6, G). When Vα7.2^+^ TCR frequencies and T-cell bleomycin-induced cell death rates were correlated, 2-dimensional plotting resulted in a distinct segregation of *LIG4*-mutated patients P1, P3, and P4 with healthy control and also with unrelated patients who had immune disease (Fig 6, H). When the slope of (% bleomycin-induced cell death)/(% Vα7.2^+^) was computed for each individual, this T-cell functional index distinctly differentiated patients with LIG4 mutation from all other individuals examined (Fig 6, H). Subset-level analysis of bleomycin-induced cell death in CD4^+^ T cells showed for naive CD4^+^ T cells a notable acceleration (Fig E6, A in this article’s Online Repository at www.jacionline.org). This was in keeping with the low *ex vivo* frequencies of this subset because naive CD4^+^ T-cell frequencies were lower and central memory CD4^+^ T-cell frequencies were reciprocally higher in P1-P4 compared with examined healthy and disease controls (Fig E6, B–D).

In summary, accelerated DNA damage-induced T-cell death is a common feature in the currently identified patients with heterozygous LIG4 R580Q and A842D monoallelic mutation.

### LIG4 R580Q and A842D mutations are functionally haploinsufficient

**FIG 7.**
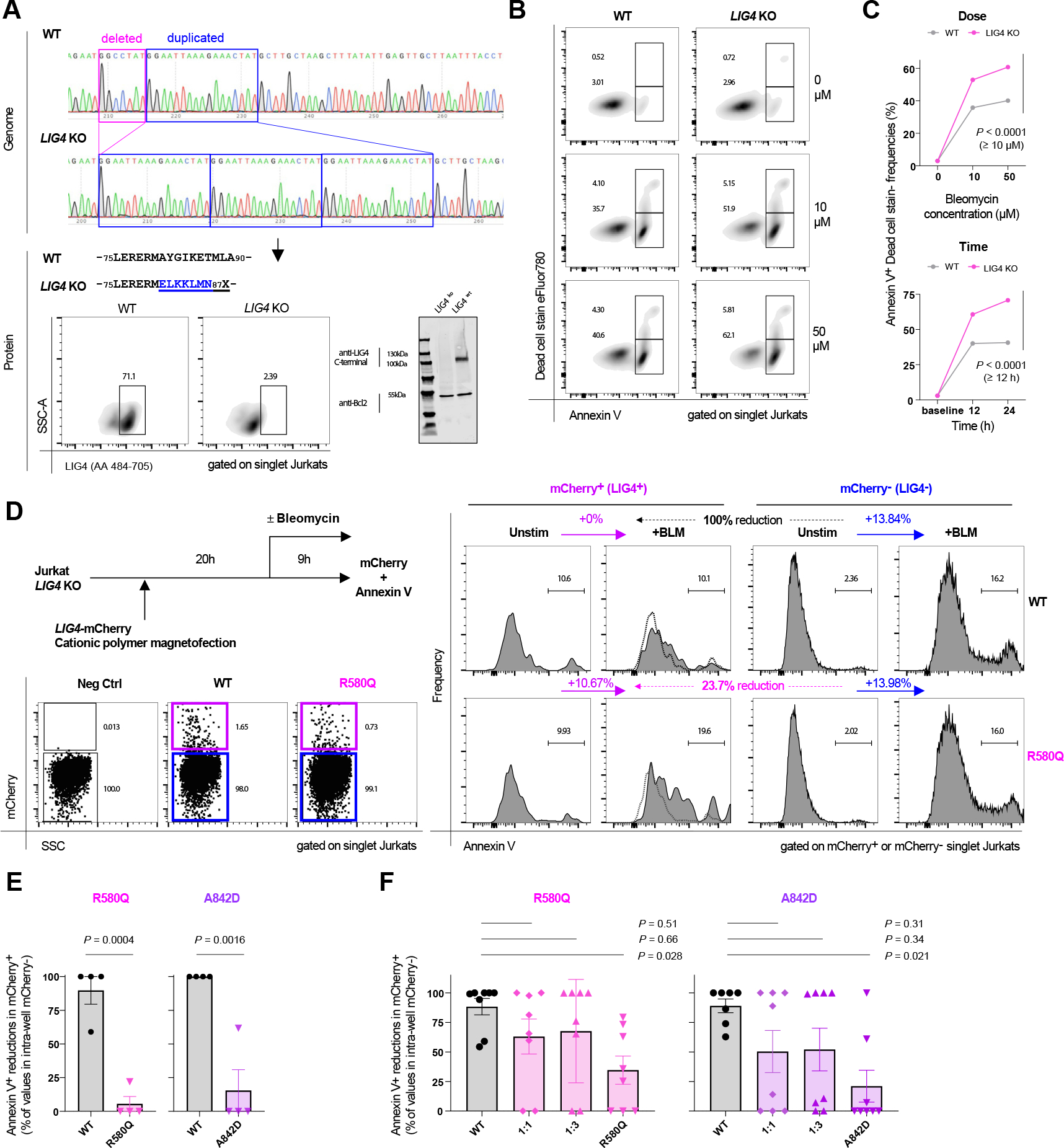
LIG4 R580Q and A842D loss-of-function mutants manifest haploinsufficiency on reconstitution. **(A)** Verification of CRISPR-Cas9-mediated *LIG4*-KO in Jurkats (*top*). LIG4-expression impairment was verified by intra-cellular staining (*bottom left*) and Western blotting (*bottom right*). **(B)** Flow-cytometric plots of WT (*left*) versus LIG4-KO (*right*) Jurkat T-cells exposed to bleomycin (12 hours). **(C)** Dose-dependent (50 μmol/L) and time-dependent (12 hours) frequencies of Annexin V–positive apoptotic cell frequencies following bleomycin exposure. Performed in triplicate (0 μmol/L, 10 μmol/L) or quadruplicate (50 μmol/L) and compared by unpaired *t*-tests. **(D)** LIG4 functional reconstitution schematic via transient overexpression in LIG4-KO Jurkat T-cells. Cells were magnetofected via cationic polymers with a dual-promoter, LIG4/mCherry coexpressing vector (representative flow plot:*bottom*), then exposed to bleomycin and evaluated for Annexin V–positivity in mCherry(/LIG4)-positive/negative populations. A representative calculation is shown. **(E)** Comparison of postbleomycin survival rates in mCherry+ cells normalized against intrawell mCherry−fractions on WT versus mutant *LIG4* transfection. Representative of 2 independent experiments performed in quadruplicate. Compared by unpaired *t*-tests. **(F)** Comparison of postbleomycin incubation survival rates in mCherry+ cells on WT and mutant LIG4 cotransfection at indicated ratios. *Post hoc* comparisons of 1-way ANOVA are shown. Pooled data of 2 independent experiments performed in triplicate/quadruplicate/control are shown (mean ± SEM). +*BLM*, Bleomycin-treated; *Neg Ctrl*, negative control; *SSC-A*, side scatter area; *Unstim*, unstimulated.

We next addressed the T-cell–intrinsic consequences of the LIG4 R580Q and A842D mutations by reconstituting LIG4 in a newly generated *LIG4*-knockout (*LIG4*-KO) Jurkat T-cell line. Using the CRISPR-Cas9 system, we generated Jurkat T cells carrying a frameshift mutation in the *LIG4* gene resulting in LIG4 loss of expression as confirmed by Western blot and flow cytometry (Fig 7, A). Bleomycin treatment of *LIG4*-KO Jurkat T cells resulted in augmented apoptosis in a dose- and time-dependent manner as compared to LIG4-competent cells (Fig 7, B and C), functionally verifying that tolerance toward DNA damage is LIG4-dependent.

We next designed a transient transfection-/overexpression-based LIG4 reconstitution in the *LIG4*-KO Jurkat T cells (Fig 7, D, top left). A combined usage of a cationic polymer with magnetofection reproducibly attained reporter protein–/LIG4 protein–positive populations (Fig 7, D, left bottom). This occurred with a low basal cytotoxicity, enabling quantitative analysis on *in vitro* DNA damage induced by bleomycin. WT LIG4-expressing Jurkat T cells typically demonstrated a rescue from cell death that was not observed in R580Q and A842D LIG4 reconstituted cells (Fig 7, D and E). There was certain interassay variability in these complex reconstitution experiments, whereas genotype differences (WT vs mutant) were consistent. Thus, both LIG4-mutant proteins are loss of function in this reconstitution system.

A mixed reconstitution of WT and R580Q or A842D LIG4 did not significantly alter T-cell apoptosis compared to reconstitution with WT alone (Fig 7, F), even when using a 3:1 ratio in favor of the mutant LIG4. These results rule out a dominant negative function of the R580Q and the A842D LIG4 variants.

In summary, the LIG4 R580Q and A842D mutations are loss of function causing LIG4 haploinsufficiency on DNA damage when present in heterozygous state.

## DISCUSSION

The clinical phenotype of human LIG4 deficiency is broad, ranging from asymptomatic carriers to death *in utero* (Table I). To our knowledge, all LIG4-deficient patients described so far carried homozygous or compound heterozygous *LIG4* mutations. However, Rucci et al^47^ described reduced survival in mice carrying a heterozygous *Lig4* missense mutation. The immune-phenotype and clinical status of parents or siblings of published patients with LIG4-deficiency has not been studied systematically yet, albeit collective experience suggests immunocompetence in those monoallelic LIG4^mut^ carriers.

All 4 patients with monoallelic novel *LIG4* mutations characterized here had hypogammaglobulinemia, low naive CD4^+^ T cells, and low Vα7.2 TCR segment usage and displayed augmented T-cell intrinsic cell death on bleomycin exposure. T-cell intrinsic hypersensitivity to experimental DNA damage in the 4 heterozygous *LIG4* mutation carriers analyzed here is a key characteristic in LIG4 deficiency.^74^

The diversified TCR repertoire in both heterozygous *LIG4* mutation carriers analyzed is in keeping with TCR repertoire analysis of published patients with compound heterozygous *LIG4* mutations.^75–78^ These similarities between published biallelic and the here-presented monoallelic *LIG4* mutation carriers might be explained by the degree of functional hypomorphism.^74^ However, this has not been studied so far. Besides the role for LIG4 in thymic T-cell development, resting peripheral T cells have been found to be particularly sensitive to DNA damage,^79^ possibly contributing to the observed low naive T cell frequencies in heterozygous *LIG4* mutation carriers.

We have documented immunodeficiency, lymphoproliferation, and autoimmunity in the patients analyzed here, including unique complications not yet documented in association with *LIG4* deficiency. However, the full clinical spectrum associated with LIG4 haploinsufficiency is predicted to widen as more patients are identified.^80,81^ We can currently not make a conclusion about the clinical penetrance of LIG4 haploinsufficiency. Penetrance and also clinical phenotypes are known to be modified by environmental influence (eg, immune-suppressive treatment or recurrent x-ray based imaging in P1), epigenetics, and rare germline variants in other immune-system genes.^82^

Our newly established transfection platform to test functionality of identified rare *LIG4* variants, in combination with molecular dynamic simulations, may guide definitive molecular diagnosis in possible LIG4 haploinsufficiency.

In summary, this is to our knowledge the first report of LIG4 haploinsufficiency associated with monoallelic *LIG4* mutations, driving human immune-dysregulatory disease that may segregate as an autosomal dominant trait. In patients with immune-dysregulation of unknown cause, we encourage physicians to consider LIG4 haploinsufficiency because it may have specific prognostic and therapeutic consequences.

## Supplementary Material

Supplemental data

Supplemental Figure E1

Supplemental Figure E2

Supplemental Figure E3

Supplemental Figure E4

Supplemental Figure E5

Supplemental Figure E6

## Figures and Tables

**TABLE I.** Clinical and genetic features of published patients with confirmed *LIG4* mutation

Mutation allele 1	Mutation allele 2	Protein 1	Protein 2	Mutational state	Additional mutations	Immune dysregulation	References	Ref ID

c.C8T + c.C26T	c.273613delC	p.A3V + p.T9I	NA	Comp het	Additional polymorphisms in *ATM, NOD2, NLRP3*	None	4	R_001
c.C32G	c.T1236T	p.A11G	p.N412K	Comp het		None	5	R_002
c.C32G	c.T1236T	p.A11G	p.N412K	Comp het	Brother with p.A11G/c.C32G, p.N412K/c.T1236T	None	5	R_003
c.C32G	c.T1236T	p.A11G	p.N412K	Comp het	Brother with p.A11G/c.C32G, p.N412K/c.T1236T	None	5	R_004
c.T57G	c.1904delA	p.L19W	p.K635fs*10X	Comp het		None	6	R_005
c.597_600delTCAG	c.597_600delTCAG	p.Q200Kfs*201	p.Q200Kfs*201	Homo		None	7	R_006
c.597_600delTCAG	c.597_600delTCAG	p.Q200Kfs*201	p.Q200Kfs*201	Homo		None	7	R_007
c.597_600delTCAG	c.597_600delTCAG	p.Q200Kfs*201	p.Q200Kfs*201	Homo		None	7	R_008
c.613delT	c.1904delA	p.S205Lfs*29X	p.K635fs*10X	Comp het		Generalized erythema and dry cracked skin	8, 9	R_009
c.613delT	c.C845A	p.S205Lfs*29X	p.H282L	Comp het	Balanced translocation t(1;19)(q21;p13))	Hepatomegaly; skin scaly, dry, and pale; hair was dry, brittle, and scarce	10	R_010
c.613delT	c.C845A	p.S205Lfs*29X	p.H282L	Comp het	Balanced translocation t(1;19)(q21;p13))	NA	10	R_011
c.613delT	c.C2440T	p.S205Lfs*29X	p.R814X	Comp het		None	8, 11, 12	R_012
c.A745G	c.1270_1274delAAAGA	p.M249V	p.K424Rfs*20X	Comp het		None	13	R_013
c.A745G	c.1271_1275delAAAGA	p.M249V	p.K424Rfs*20X	Comp het		Jaundice, sclerosing cholangitis, hepatosplenomegaly	5	R_014
c.A745G	c.1271_1275delAAAGA	p.M249V	p.K424Rfs*20X	Comp het		Jaundice, sclerosing cholangitis, hepatosplenomegaly	5	R_015
c.G827A	c.233_236delAGAG	p.G276D	p.R78Wfs*15X	Comp het		Disseminated erythematous maculopapules after rubella vaccine, hepatosplenomegaly	14	R_016
c.G833A	c.G833A	p.R278H	p.R278H	Homo		NA	15	R_017
c.G833A	c.G833A	p.R278H	p.R278H	Homo		Hypopigmentation, bronchiectasis	6	R_018
c.G833A	c.G833A	p.R278H	p.R278H	Homo		Hypopigmentation	6	R_019
c.G833A	c.G833A	p.R278H	p.R278H	Homo	For all 3 mutations + p.A3V + p.T9I/c.C8T + c.C26T	None	16–19	R_020
c.G833A	c.G833A	p.R278H	p.R278H	Homo		None	16, 20, 21	R_021
c.G833A	c.1271_1275delAAAGA	p.R278H	p.K424fs*20X	Comp het		NA	22	R_022
c.G833A	c.1271_1275delAAAGA	p.R278H	p.K424fs*20X	Comp het		NA	15	R_023
c.G833A	c.1271_1275del	p.R278H	p.K424Rfs*21X	Comp het		NA	51	R_024
c.G833T	c.G833T	p.R278L	p.R278L	Homo		None	23, 78	R_025
c.G833T	c.G833T	p.R278L	p.R278L	Homo		None	21	R_026
c.G833T	c.935delC	p.R278L	p.P313Hfs*19	Homo		AIHA	23, 78	R_027
c.G833T	c.1142_1143delCT	p.R278L	p.L382Efs*4	Comp het	c.C26T/p.T9I	AIHA	23, 78	R_028
c.G833T	c.1144_1145delCT	p.R278L	p.L382Efs*5	Comp het		Gastrointestinal ulcers	24	R_029
c.G833T	c.1271_1275delAAAGA	p.R278L	p.K424Rfs*20X	Comp het		Vitiligo	24	R_030
c.G833T	c.1271_1275delAAAGA	p.R278L	p.K424Rfs*20X	Comp het		Erythroderma	24	R_031
c.G833T	c.1271_1275delAAAGA	p.R278L	p.K424Rfs*20X	Comp het		Eczema, generalized lymphadenopathy	24	R_032
c.G833T	c.1271_1275delAAAGA	p.R278L	p.K424Rfs*20X	Comp het		None	24	R_033
c.G833T	c.1271_1275delAAAGA	p.R278L	p.K424Rfs*20X	Comp het		None	24	R_034
c.G833T	c.1271_1275delAAAGA	p.R278L	p.K424Rfs*20X	Comp het		None	23, 78	R_035
c.G833T	c.1271_1275delAAAGA	p.R278L	p.K424Rfs*20X	Comp het		Colitis	23, 78	R_036
c.G833T	c.1271_1275delAAAGA	p.R278L	p.K424Rfs*20X	Comp het		AIHA, purpura	78	R_037
c.G833T	c.1271_1275delAAAGA	p.R278L	p.K424Rfs*20X	Comp het		None	78	R_038
c.G833T	c.1271_1275delAAAGA	p.R278L	p.K424Rfs*20X	Comp het		AIHA	78	R_039
c.G833T	c.1271_1275delAAAGA	p.R278L	p.K424Rfs*20X	Comp het		Anti-human globulin test, antithrombocyte antibodies, anti-HLA antibodies	25	R_040
c.G833T	c.1277_1278delAA	p.R278L	p.E426Gfs*19	Comp het		None	24	R_041
c.G833T	c.G2113T	p.R278L	p.E705X	Comp het		None	23, 78	R_042
c.G833T	c.2134_2135delTA	p.R278L	p.I712Afs*5	Comp het		AIHA	23, 78	R_043
c.G833T	c.C2710T	p.R278L	p.Q904X	Comp het	p.S12T/c.T34A	None	78	R_044
c.G833T	Loss exon2 (189–4043)	p.R278L	None	Comp het		None	78	R_045
c.G833C	NA	p.R278P	p.E582Dfs	Comp het		None	26	R_046
c.A840G	c.1271_1275delAAAGA	p.Q280R	p.K424Rfs*20X	Comp het	No AV3, T9I	None	19, 77	R_047
c.A840G	c.1271_1275delAAAGA	p.Q280R	p.K424Rfs*20X	Comp het	No AV3, T9I	None	19, 77	R_048
c.A845T	c.1544_1548delAAAGA	p.H282L	p.K424Rfs*19X	Comp het		Veno-occlusive disease	19, 76	R_049
c.A845T	c.1544_1548delAAAGA	p.H282L	p.K424Rfs*19X	Comp het		Autoimmune cytopenia	19, 76	R_050
c.C845T	c.1746_1750delAAGAT	p.H282L	p.R581fsX	Comp het	c.C26T/p.T9I	Omenn syndrome (scaly erythroderma), hepatosplenomegaly, lymphadenopathy	19, 27	R_051
c.C847G	c.C847G	p.K283E	p.K283E	Homo		NA	28	R_052
c.A847A	c.1271_1275delAAAGA	p.K283E	p.K424Rfs*20X	Comp het		NA	29	R_053
c.A847A	c.1271_1275delAAAGA	p.K283E	p.K424Rfs*20X	Comp het		None	29	R_054
c.A875G	c.1307_1311del	p.Q229R	p.K436Rfs*20	Comp het		NA	51	R_055
c.G907A	c.1904delA	p.P231T	p.A562fs21X	Comp het		None	30	R_056
c.T980G	c.2585_5886del	p.I327S	p.H826Rfs*6	Comp het		AIHA	78	R_057
c.G1102T	c.G1102T	p.D368Y	p.D368Y	Homo		Eczema	31	R_058
c.A1103T	c.G1341T	p.D368V	p.W447C	Comp het		Bronchiectasis, villous atrophy, liver lesions, granulomatous dermatitis (after rubella vaccination, nodular, superficial and deep dermal lymphohistiocytic infiltrate with scattered lymphohistiocytic cells)	32	R_059
c.G1237T	c.G1341	p.E413*	p.W447C	Comp het		Epithelioid cell granuloma (absence of infection)	19, 33	R_060
c.1245_1250dupGATGC	c.C2440T	p.L418Mfs*3	p.R814X	Comp het		None	9	R_061
c.1271_1274delAAAG	c.C2440T	p.K424Rfs*20X	p.R814X	Comp het		NA	51	R_062
c.1271_1275delAAAGA	c.C2440T	p.K424Rfs*20X	p.R814X	Comp het		Psoriasis	9	R_063
c.1271_1275delAAAGA	c.C2440T	p.K424Rfs*20X	p.R814X	Comp het		None	9	R_064
c.1271_1275delAAAGA	c.C2440T	p.K424Rfs*20X	p.R814X	Comp het		None	9	R_065
c.1271_1275delAAAGA	c.C2440T	p.K424Rfs*20X	p.R814X	Comp het		Hypopigmentation	9	R_066
c.1271_1275delAAAGA	c.C2440T	p.K424Rfs*20X	p.R814X	Comp het		None	9	R_067
c.1271_1275delAAAGA	c.C2440T	p.K424Rfs*20X	p.R814X	Comp het		None	9	R_068
c.1271_1275delAAAGA	c.C2440T	p.K424Rfs*20X	p.R814X	Comp het		None	29	R_069
c.1271_1275delAAAGA	c.C2440T	p.K424Rfs*20X	p.R814X	Comp het		Cutaneous abnormalities	28	R_070
c.A1296T	c.C1672T	p.K432N	p.Q558X	Comp het		None	78	R_071
c.1297_1299delCAA	c.1297–1299delCAA	p.Q433del	p.Q433del	Homo		None	19, 34	R_072
c.T1312c	c.T1312c	p.Y438H	p.Y438H	Homo	LRIG2 mutations (homo)	Nail dystrophy, sparse and thin hair	35	R_073
c.A1345C	c.C2440T	p.K449Q	p.R814X	Comp het		None	75	R_074
c.A1345C	c.C2440T	p.K449Q	p.R814X	Comp het		None	75	R_075
c.A1345C	c.C2440T	p.K449Q	p.R814X	Comp het		None	75	R_076
c.A1345C	c.C2440T	p.K449Q	p.R814X	Comp het		NA	15	R_077
c.G1406A	c.C2440T	p.G469E	p.R814X	Comp het		Psoriasiform erythrodermic squamous skin patches	17, 18, 36	R_078
c.G1406A	c.C2440T	p.G469E	p.R814X	Comp het		None	37	R_079
c.1512_1513delTC	c.C2440T	p.R505Cfs*12X	p.R814X	Comp het		None	9	R_080
c.1751_1755delTAAGA	c.C2440T	p.I584Rfs*2X	p.R814X	Comp het		None	38	R_081
c.1762delAAG	c.1762delAAG	p.K588del	p.K588del	Homo		None	39	R_082
c.1762delAAG	c.1762delAAG	p.K588del	p.K588del	Homo		None	39	R_083
c.C1738T	c.C2440T	p.R580X	p.R814X	Comp het		Hypothyroidism, hypogonadism, diabetes, chronic cutaneous affection, photosensitivity, telangiectasia	17	R_084
c.C1738T	c.C2440T	p.R580X	p.R814X	Comp het		Hypothyroidism, amenorrhea, photosensitivity, psoriasis	17	R_085
c.1904delA	c.C2440T	p.K635fs*10X	p.R814X	Comp het		NA	28	R_086
c.1904delA	c.C2440T	p.K635fs*10X	p.R814X	Comp het		NA	28	R_087
c.C2094T	c.C2440T	p.Y698X	p.R814X	Comp het		None	9	R_088
c.C2094T	c.C2440T	p.Y698X	p.R814X	Comp het	Xp22.31p22.32 duplication	None	40	R_089
c.2386 2389dupATTG	c.C2440T	p.A797Dfs*3	p.R814X	Comp het		Cutis marmorata	9	R_090
c.C2440T	c.C2440T	p.R814X	p.R814X	Homo		Hypogonadism, asthma, lymphadenopathy, hepatomegaly	41	R_091
c.G2612A	c.G2612A	p.R871H	p.R871H	Homo		Recurrent meningitis (sterile), recurrent genital/oral ulcers, anterior uveitis, intermittent attacks of nonerosive arthritis	42	R_092
NA	NA	NA	NA	NA	AML: 48, XX, 12, der(5) t(5;17)(q11;q11), −7, 18, 111, −17, 120/46, XX	None	43	R_093
NA	NA	NA	NA	NA		None	19	R_094
NA	NA	NA	NA	NA		Autoimmunity, Omenn phenotype	19	R_095
NA	NA	NA	NA	NA		None	19	R_096
NA	NA	NA	NA	NA		None	19	R_097
NA	NA	NA	NA	NA		None	19	R_098
NA	NA	NA	NA	NA		None	19	R_099
NA	NA	NA	NA	NA		None	19	R_100
NA	NA	NA	NA	NA		None	19	R_101
NA	NA	NA	NA	NA		None	19	R_102
NA	NA	NA	NA	NA		None	19	R_103
NA	NA	NA	NA	NA		None	19	R_104
NA	NA	NA	NA	NA		None	19	R_105
NA	NA	NA	NA	NA		None	19	R_106
NA	NA	NA	NA	NA		Autoimmunity	19	R_107
NA	NA	NA	NA	NA		None	19	R_108
NA	NA	NA	NA	NA		None	19	R_109
NA	NA	NA	NA	NA		None	19	R_110
NA	NA	NA	NA	NA		None	19	R_111
NA	NA	NA	NA	NA		None	19	R_112
NA	NA	NA	NA	NA		None	19	R_113
NA	NA	NA	NA	NA		None	19	R_114
NA	NA	NA	NA	NA		None	19	R_115
NA	NA	NA	NA	NA		None	19	R_116
NA	NA	NA	NA	NA		None	19	R_117
NA	NA	NA	NA	NA		NA	19	R_118
NA	NA	NA	NA	NA		NA	19	R_119
NA	NA	NA	NA	NA		NA	19, 44	R_120

*AIHA,* Autoimmune hemolytic anemia; *AML,* acute myeloid leukemia; *Comp het,* compound heterozygous; *Homo,* homozygous; *NA,* not available; *XX,* 2 x-chromosomes. Patients are ordered according to the 5′ position of the first mutated allele. cDNA sequence refers to NM_001098268.

## Clinical implications:

LIG4 haploinsufficiency should be considered in patients with immune dysregulation of unidentified cause, because it may have prognostic as well as therapeutic consequences.

## Abbreviations used

BCR: B-cell receptor
BRCT2: BRCA1 C-terminal domain 2
DSB: DNA double-strand break
HD: Healthy donor
IGH: Immunoglobulin heavy chain
IGHA: Immunoglobulin heavy constant α
IGHG: Immunoglobulin heavy constant γ
IR: Ionizing radiation
KO: Knockout
MD: Molecular dynamics
MD memory: IgM/IgD memory B cells
NHEJ: Nonhomologous end-joining
P1: Patient 1
TCR: T-cell receptor
TCRA: T-cell receptor α-chain
V(D)J: Variable, diversity, and joining gene segments
WT: Wild type
