# Supplementary material for: Autoimmunity and immunodeficiency associated with monoallelic LIG4 mutations via haploinsufficiency
Source: J Allergy Clin Immunol. Author manuscript; Available in PMC 2024 Feb 1. (PMC10529397; doi:10.1016/j.jaci.2023.03.022)
Supplement: Supplemental data [file NIHMS1915164-supplement-Supplemental_data.docx]

**Supplemental data**

## **Supplemental methods**

*Genetic analysis, variant calling, annotation and filtering*

# Genetic sequencing was performed following informed consent. Genomic DNA was isolated from cultured T-cell blasts or PBMCs using the QIAamp DNA Blood Mini Kit (Qiagen). Whole exome sequencing was performed as described earlier^(1, 2)^. The fragmented DNA was hybridized with exon-specific biotinylated primers (SureSelect Human All Exon V6, Agilent Technologies), Dynabeads (MyOne Streptavidin T1 magnetic beads, Thermo Fisher Scientific) were used for the exons pull-down. Paired-end 125bp sequencing was completed on Illumina HiSeq 4000 platform (Functional Genomics Center - Zurich, Switzerland). For each sample, the paired-end sequencing resulted in two FASTQ files (fwd and rev). Those were subjected to quality control and aligned to the reference genome (GCRCh37 - hg19) using Burrows-Wheeler aligner, which generated sequence alignment map files. Picard tools (v.2.7.1) was used to make a binary version of the sequence alignment map files. Quality control and genotype calling were performed using the Genome Analysis Toolkit (gatk, Broad Institute, USA)^(3)^ and variants were annotated with the position of nucleotide change with respect to coding genes. HaplotypeCaller from the GATK was utilized for variant calling, resulting in a variant called file. CADD (v.1.3) and vVariant effect predictor programs were used for mutation annotation. To elucidate a common disease-driving variant in P1 and her father, variants with coverage of < 10 were excluded, variants encoding synonymous or inframe- mutations were excluded, further shared variants with the mother and brother were excluded, the only mutation which was common in the father and P1 was *LIG4*.

The *LIG4* variant was confirmed by Sanger sequencing of PCR amplification products of cDNA derived from PBMCs. After running the amplicon on a 1.5% agarose gel, DNA was extracted with QIAquick Gel Extraction Kit (Qiagen). The purified PCR products were then bidirectionally sequenced by Microsynth (Switzerland).

*Histology and radiology*

For P1 all the available histological specimens, immunohistochemical stains and radiological investigations were reviewed and performed as part of the clinical routine

*Blood samples routine phenotyping, immunoglobulin quantification and ALPS biomarkers*

Differential white blood cell counts were obtained from all IEI patients. For patient P1 T and B cells were immunophenotyped during the clinical routine (surface markers for B and T cell subsets phenotyping are found in table X). Serum levels of total IgG, IgG subclasses, IgM and IgA were measured by the ISO 17025 accredited Medical Immunological Laboratory of the University Hospital Basel. The ALPS biomarkers (Vitamin B12, sFASL) were tested as in the clinical routine laboratory at the University Hospital Basel and the Center for Chronic Immunodeficiency in Freiburg, Germany.

*Cell isolation and in vitro T cell activation*

IEI-patients, their realtives and healthy control-derived peripheral blood mononuclear cells (PBMCs) were isolated from whole blood, via Ficoll density gradient separation using Lymphoprep^TM^ (density of 1.077g/mL, Axonlab).

PBMCs derived T cell blasts were expanded with initial phytohaema­glutinin stimulation (5g/ml, Sigma Aldrich) and human IL-2 (300U/ml, Proleukin®, Novartis), cultured in media RPMI (Sigma-Aldrich) supplemented with 10% heat-inactivated FCS (v/v, Gibco), 1% penicillin-streptomycin (v/v, Gibco), 1% none-essential amino acids (v/v, Gibco) and 2mM Glutamax (v/v, Gibco) at 37°C in a humified 5% CO_2_ incubator. IL-2 was renewed every 5-7 days.

Cell activation: PBMCS, either freshly isolated or thawed from liquid nitrogen-stored samples, were cultured in RPMI with 5% human AB-serum (blood donation center Basel, Switzerland), 1mM Sodium Pyruvate (Gibco), 1% penicillin-streptomycin (Gibco), non-essential amino acids (Gibco) and 1% Glutamax (Gibco). T cells were stimulated with anti-CD3 ([1mg/mL], OKT3, Ultra-LEAF^TM^, BioLegend # 317325) and anti-CD28 ([2mg/mL], 28.2, Ultra-LEAF^TM^, BioLegend # 302933), at a cell concentration of 1^6^/mL.

For evaluation of T cell proliferation, CellTrace^TM^ Violet Cell Proliferation kit (0.5μM, ThermoFisher, # C34557) was used for PBMCs labeling. Cell concentration was adjusted to 10^6^/mL, then incubated at 37°C in the dark for 20min, followed by 10min incubation on ice. 10 volumes of media were added, followed by 2 washing steps. Cells were stimulated as described above for five consecutive days.

For the in vitro apoptosis assay PHA T cells blasts were incubated four hours with either anti-CD3 (BioLegend, Ultra-LEAF^TM^ OKT3 #317325, 0.5 - 5μg/mL), anti-Fas (BioLegend, Ultra-LEAF^TM^ EOS9.1 #305705, 0.5 - 5μg/mL), recombinant FasL (eBioscience, NOK-1 #16-9919-81, 0.5 - 5μg/mL) or staurosporine (Sigma #S5921, 0.1 – 0.5μM). The surface staining and the AnnexinV-binding coupled to fluorochrome (BioLegend, #640905) were performed in AnnexinV binding buffer (BioLegend, #422201) for 30min at RT.

*Immunophenotyping*

Staining for cell surface markers on PBMCs was performed with the following fluorochrome-conjugated antibodies to the indicated proteins

| 1° antibodies | | | | |
| --- | --- | --- | --- | --- |
| Antigen | fluorochrome | clone | Dilution | Reference |
| CCR6 | BV421^TM^ | G034E3 | 50 | BioLegend # 353407 |
| CD3 | BV711^TM^, PE  PE-Cy7, PB,  BUV395 | OKT3  UCHT1 | 20 | BioLegend # 317327, 317307  BioLegend # 300419, 300418  BD # 563548 |
| CD4 | Ax700,  BV510^TM^ | OKT4  SK3 | 40 | BioLegend # 317425,  344634 |
| CD8 | PE/Dazzle^TM^, APC  BUV395 | SK1  HIT8a | 40 | BioLegend # 344743, 344721  BD # 740303 |
| CD19 | Ax700 | HIB19 | 40 | BioLegend # 302225 |
| CD20 | BV510^TM^, PE/Cy5 | 2H7 | 40 | BioLegend # 302339, 302307 |
| CD21 | BV711^TM^ | 1048 | 50 | BD Bioscience #742763 |
| CD25 | Ax488 | BC69 | 50 | BioLegend # 302615 |
| CD27 | BV650^TM^ | O323 | 50 | BioLegend # 302827 |
| CD38 | APC | HIT2 | 50 | BioLegend # 303509 |
| CD45RA | FITC | HI100 | 50 | BioLegend # 983002 |
| CD45R0 | BV510^TM^ | UCHL1 | 50 | BioLegend # 304245 |
| CD69 | BV421^TM^, BV605^TM^ | FN50 | 50 | BioLegend # 310929, 310937 |
| CD127 | BV650^TM^,  BV711^TM^, PE/Cy7 | A019D5 | 50 | BioLegend # 351325, 351327, 351320 |
| CD152/CTLA-4 | PE, BV421^TM^ | BNI3 | 30 | BD # 557301, 562743 |
| CD137/4-1BB | PE/Cy7 | 4B4-1 | 50 | BioLegend # 309818 |
| CD161 | BV605^TM^ | HP-3G10 | 20 | BioLegend # 339915 |
| CD183/CXCR3 | Alexa Fluor 647 | G025H7 | 50 | BioLegend # 353711 |
| CD185/ CXCR5 | BUV395 | RF8B2 | 50 | BD # 740266 |
| CD194/CCR4 | PE-Cy7 | 1G1 | 50 | BD # 557864 |
| CD196/CCR6 | PE | 11A9 | 50 | BD # 551773 |
| CD223/ LAG-3 | APC | 7H2C65 | 100 | BioLegend # 369211 |
| CD279/ PD-1 | BV785 ^TM^ | EH12.2H7 | 100 | BioLegend # 329929 |
| CD366/Tim-3 | BV605 ^TM^ | F38-2E2 | 50 | BioLegend # 345018 |
| IgD | PE-Cy7 | IA6-2 | 50 | BioLegend # 348209 |
| IgM | BV605 ^TM^ | MHM-88 | 100 | BioLegend # 314523 |
| Ig light chain κ | FITC | MHK-49 | 50 | BioLegend # 316507 |
| Ig light chain λ | PE | MHL-38 | 50 | BioLegend # 316607 |
| TCRαβ | Alexa Fluor 488 | IP26 | 40 | BioLegend # 306711 |
| TCRγδ | PE | B1 | 20 | BioLegend # 331209 |
| Vα7.2/ Jα33 | PE | 3C10 | 20 | BioLegend # 351705 |
| 2°Antibodies | | | | |
| Goat anti-Rabbit IgG (H+L), min X Hu Sr Prot | Alexa Flour 488 | Polyclonal | 500 | Jackson ImmunoResearch # 111-545-045 |
| Goat anti-Rabbit IgG (H+L), min X Hu Sr Prot | Alexa Fluor 647 | Polyclonal | 500 | Jackson ImmunoResearch #  111-605-045 |

Staining media: Cells were stained in PBS containing 2.5% human AB serum, NaH_3_ 0.01%, Hepes 25mM and Fc block (TrueStain, BioLegend # 426101) for 30min at 4°C. Chemokine receptor staining was performed at 37°C for 20 min.

Cell viability was assessed using Live/Dead Fixable NIR (# L34975, Invitrogen^TM^, ThermoFisher

Scientific). Data were acquired on an LSRII-Fortessa ^TM^ (BD Bioscience – equipped with 355nm, 405nm, 488nm, 561nm and 640nm laser lines) for the R580Q carriers and on acquired on the CytoFLEX S (Beckman Coulter) for the A842D carriers. Before each acquisition, the photomultiplier (PMT) voltages were manually adjusted with single stain controls to minimize fluorescence spillover and calculation of compensation matrixes. Data analysis was performed using FlowJo software (Version 10.5.2, TreeStar, USA).

*Intracellular cytokine production by T cells*

For intracellular cytokine production, we incubated PBMCs for 4-5 hours at 37°C in media with PMA (50ng/mL, Sigma), ionomycin (500ng/mL Sigma) and BrefeldinA (BioLegend, 5ug/mL). Cells were first stained for surface marker and viability, then fixed and permeabilized using the BD Cytofix/Cytoperm^TM^ (BD Bioscience), next intracellular staining was performed using BD Perm/Wash Buffer (BD Bioscience).

| Antigen | fluorochrome | clone | Dil. | Reference |
| --- | --- | --- | --- | --- |
| IL-2 | APC | MQ1-17H12 | 50 | BioLegend # 500311 |
| IL-17A | PE | BL168 | 50 | BioLegend # 512305 |
| INF-γ | BV421^TM^ | 4S.B3 | 50 | BioLegend # 502531 |
| TNF-α | BV421^TM^, PE-Cy7 | Mab11 | 50 | BioLegend # 502931, 502929 |

*Intranuclear transcription factor and phospho-protein staining*

The expression of transcription factors was determined by intranuclear staining performed according to the manufacturers’ Transcription Factor staining buffer protocols (eBioscience^TM^).

For the detection of phosphor-proteins, PBMCs were fixed and permeabilized in BD Cytofix/ Cytoperm^TM^ (BD Bioscience) for 20min at room temperature (RT), next the cells were permeabilized with BD Perm III for 30min at 4°C. Primary anti-phospho antibodies were added in staining media for 1h at RT, after washing secondary antibodies were added subsequently for 30min at RT.

| Antigen | fluorochrome | clone | Dil. | Reference |
| --- | --- | --- | --- | --- |
| FoxP3 | PE | 150D | 20 | BioLegend # 320007 |
| γH2Ax (Ser139) | Alexa Fluor488 | 2F3 | 20 | BioLegend # 613405 |
| phospho-53BP1 (Ser1778) | unconjugated | Polyclonal | 100 | Cell Signaling # 2675 |

*Radiosensitivity Testing and DNA-damaging compounds*

PBMCs from patients and healthy controls were γ-irradiated with indicated Gy dose (^137^Cs Gamma counter). Cells were harvested at indicated time-points, before flowcytometry based analysis of phospho-proteins (see above).

Bleomycin sulfate (European Pharmacopoeia EP Reference Standard, cat# B1141000) was diluted in human cell culture media and added to the PBMCs for the indicated duration.

*Immunoblotting*

Protein extracts were prepared from immune cells through lysis with RIPA buffer (Thermo Fisher Scientific) containing protease/phosphatase inhibitors (Roche). Protein denaturation was achieved with 1x Lämmli sample buffer (Bio-Rad) at 90°C for 10min. Proteins were separated by electrophoresis using 4-15% Mini Protean TGX Gel (Bio-Rad) and transferred to nitrocellulose membranes (semi-dry Trans-Blot Turbo Transfer, Bio-Rad). The membranes were incubated in blocking solution (TBST with 5% non-fat milk powder) for several hours at room temperature, followed by a 1° antibody directed against the antigen of interest in blocking solution. After washing, membranes were incubated with appropriate 2° antibodies and images were captured using LICOR Odyssey imaging system.

| Antigen | Host, isotype | clone | Dil. 1in | Reference |
| --- | --- | --- | --- | --- |
| β-Actin | Rabbit, IgG | D6A8 | 1000 | Cell Signaling Technology # 8457 |
| Bcl-2 | Rabbit, IgG | D55G8 | 1000 | Cell Signaling Technology # 4223 |
| LIG4 | rabbit IgG | D5N5N | 1000 | Cell Signaling Technology #14649 |
| 2° antibodies  Host | Reactivity | Fluorochrome | clone |  |
| Goat | rabbit IgG (H+L) | IRDye-800CW | polyclonal | LICOR: 926-32211 |
| Goat | mouse IgG (H+L) | IRDye-800CW | polyclonal | LICOR: 926-32219 |

*Generation and analysis of TCR and IgH repertoire by NGS*

Bulk T and B cells were analyzed by deep sequencing. In brief, for TCR repertoire analysis genomic DNA was extracted from PBMCs using QIAamp DNA Blood Mini Kit (Qiagen) the quantity and purity were assessed with spectrophotometric analysis. For the IgH repertoire analysis, PBMCs were lysed in RLT buffer (Qiagen).

SuperScript III/IV (Invitrogen) was utilized for reverse transcription and the IgH constant region primers included a 14nt unique molecular identifiers (UMI) and partial p7 adaptors. Per sample two individual reverse transcription were run, the first with IgM and IgD specific reverse primers and the second with IgA, IgG and IgE-specific reverse primers. Primer sequence and PCR conditions can be found in Ghraichy M., 2020^(4)^. Two-step multiplex PCR amplifications were used for IgH gene rearrangements, 1^st^ step used a mix of FR1 V family-specific primers with partial p5 adaptors, the 2^nd^ amplification step completed the adaptor sequences. Gel-based separation for PCR amplicons was performed, amplicons were gel-extracted, followed by purification and quantification (following Illumina qPCR library quantification protocol). Each library was normalized for its concentration, followed by multiplexing in batches of 24 for sequencing on the Illumina MiSeq platform (2x 300bp paired-end chemistry). IgH samples were de-multiplex with help of their Illumina indices and processed using the Immcantation platform^(5, 6)^. Briefly, filtering of raw FASTQ files was performed if the quality score was >20. Joining of paired reads was accomplished if they had a at least a 10nt length, a maximum error rate of 0.3 and the α threshold of 1^-4^. Reads with identical UMI were collapsed. Further read collapsing was performed if the reads displayed same full-length sequence and the same constant primer but different UMI– resulting in a data frame containing unique sequences per sample and isotype. VDJ assignment was performed with IgBlast^(7)^, IMGT germline database was taken as a reference for sequence annotation. The Stampy^(8)^ algorithm was used for mapping the constant region sequences to the germline, only sequences with a well-defined constant region were further analyzed. B cell populations were defined according to the constant region annotation and mutation number. V genes were classified as “unmutated” (naïve) if ≤ 2nt mutations were found across IgD and IgM sequences (correcting for sequencing bias and allelic variance). The amount and type of V gene mutations were calculated with the R package SHazaM(^6)^. SHM levels were computed by calculating V gene mutations in single sequences and average values were computed across samples and cell subsets. The SHazaM R package allowed for an effective representative sequence of each clonal group, this legitimated the computation of the selection pressure using BASELINe^(9)^. Selection was tested by calculating CDR_R/(CDR_R + CDR_S), which corrects age-dependency.

For the TRA and TRB loci analysis, rearranged products were amplified via multiplex PCR (Adaptive

Biotechnologies, USA). In short, 52 forward primers were used for TRBV gene segments and 13 reverse primers to cover the TRBJ gene segments. The amplicons were sequenced using the Illumina HiSeq platform, the assay was designed at a survey level (detection limit 1 cell in 0.04e^6^). Amplification bias due to multiplex PCR was reduced by Adaptive Biotechnologies with assay-based and computational approaches. Custom algorithms were used for the alignment of the reads to reference genome sequences. ImmunoSEQ Analyzer (V.3.0), was utilized for read processing. One clonotype was defined as the unique arrangement of a CDR3 amino acid sequence and its related V gene. Heatmaps for the visualization of TRAV to TRAJ gene pairing in total and unique sequences were produced with the Morpheus platform (Broad Institute, USA). The CDR3 self-reactivity indices were investigated, as described previously^(10)^. The TRAV gene differential expression was calculated with the R platform, using the Limma package^(11)^ (v3.36.5). Data was visualized with the ggplot2 package (version 3.3.2).

*Molecular dynamic simulations*

The atomic model of the human LIG4 catalytic domain was modeled using the SWISS-MODEL server^(12)^ and was based on the crystal structure PDB code 6BKG, assumed to correspond to the closed state of the enzyme encircling an adenylated DNA strand, resolved at 2.4 Å. The model used for the study of the A842D variant was based on the x-ray structure under the accession code 3II6.pdb. Whereas the 6BKG deposited structure already corresponds to the protein of interest (no homology), this strategy allowed to automatically build the missing loops. The longest missing loop was three residues long. The variants were introduced with VMD^(13)^. The adenylated DNA strand was built with Chimera^(14)^ and the force field parameters were generated with the CGenFF web-service as previously described^(15-17)^. The systems containing the modeled protein, either the WT or the R580Q resp. A842D, the DNA and the adenylated DNA strands, were solvated with ≈ 43,000 water molecules, represented by the TIP3 model^(18)^ and further neutralized at a salt concentration of 150 mM with K^+^ and Cl^-^ counterions. While experiments show that LIG4 requires magnesium for nick sealing, no divalent ions were observed in the crystal structure. However, metal-binding residue candidates were proposed^(19)^. We added manually magnesium ions within coordination distance (4Å) of Asp_275_, Glu_331_ and Glu_427_. Since the distance between the Cβ atom of residue 580 is located at ~18 Å of the magnesium coordinating candidates, the dynamics of the divalent ions were not further studied. We noted however that they remained coordinated by these acidic residues during the whole length of the simulations. A typical system contained ~ 140,000 atoms. Molecular dynamics simulations were conducted with the GROMACS package (v.2018^)(20)^ and the CHARMM force-field (v27)^(21)^. The angles as well as bond lengths, which involved hydrogen atoms were constrained using the LINCS algorithm^(22)^. The 1.2nm time point was chosen for the short-range electrostatics cut off, and the Ewald method for particle mesh was utilized for long-range electrostatic^(23)^. Lennard-Jones potential was used to describe the Van der Waals interactions with up to a distance of 1.2 nm. A 1 bar constant pressure was maintained utilizing the Parrinello-Rahman algorithm (time constant 5ps). The 310°K temperature was kept using the Nose-Hoover algorithm (time constant 1ps). The atomic systems representing the protein with the DNA were equilibrated following the CHARMM-GUI protocol^(24)^.

For each investigated sequence (WT or R580Q), six independent individual trajectories of 500 ns length were generated, amounting to 6 μs of simulation. Four independent trajectories per sequence, each also 500 ns long, were performed in the study of the A842D variant. Structural biology analyses were performed using an ensemble of R, python, and tcl in-house scripts. Molecular representations were generated with VMD. The free energy of interactions was calculated by solving the adaptive Poisson-Boltzmann equation(25), using the g_mmpbsa module implemented in GROMACS^(26)^.

*Recombinant LIG4 and nick sealing assay*

WT and mutant LIG4 proteins (aa 1-620) were produced by GeneScript.

To generate a nicked DNA duplex, three oligonucleotides (see below) were annealed and phosphor­ylated by mixing equimolar amounts in a buffer comprising 40mM Tris, 10mM MgCl_2_, 10mM dithiothreitol (DTT), 0.5mM ATP and PNK kinase (New England Biolabs). Oligonucleotides were phosphorylated at 37°C for 30min, then heated to 95°C for 5min with sequential cooling steps till 25°C. Ligation reactions contained the three oligonucleotides, the respective DNA ligase4 (either wt or 580Q mutant), reaction buffer was the same as for the phosphorylation/annealing of the oligonucleotides, incubation was performed at 37°C, the time of ligation is noted in the respective figure legend. Ligation reactions were quenched by the addition of an equal volume of loading buffer (95% formamide, 18mM EDTA, 0.025% SDS) for 5min at 95°C and rapid cooling on ice. Products were characterized by denaturing PAGE (15% polyacrylamide TBE-Urea) in 0.5x TBE buffer and ligation was visualized and quantified with LICOR Odyssey imaging system.

*RNA extraction and real-time PCR*

Primers were designed with NCBI Primer-BLAST. RT-PCR primers should create amplicons spanning exon to exon junctions or designed within two separate exons with an > 1000bp intron in between. Cells were lysed by adding TRIzol (Thermo Fisher Scientific), RNA was extracted from immune cells using the QIAmp RNA Blood Mini Kit (Qiagen). RNA content was measured by NanoDrop Spectrophotometer (Thermo Fisher Scientific). Consequently, the first-strand cDNA was generated via GoScript^TM^ Reverse Transcription System (according to the manufacturer’s protocol Promega) using random hexamer primers and 3mM MgCl_2_. RT-PCR reactions contained variable amounts of template cDNA. PCR amplification was carried out in triplicates using the GoTaq qPCR reagents (Promega), amplification was done using Real-Time PCR cycler Vii A7 (ThermoFisher Scientific). PCR conditions were 40 cycles à 30s - 95°C, 1 min - 61°C. Transcript levels were normalized for the housekeepers ACTB, GAPDH, PGK1 and HRPT1 and subsequently to healthy controls, using the equation by W.Pfaff^l(27)^.

$$\frac{{{(E}_{target})}^{{\Delta CP}_{{target}^{(control-sample)}}}}{{{(E}_{reference})}^{{\Delta CP}_{{reference}^{(control-sample)}}}}$$

*Oligonucleotides*

Oligonucleotides were ordered with Microsynth (Switzerland).

|  | Forward 5’-3’ | Reverse 5’-3’ | Source |
| --- | --- | --- | --- |
| Sanger |  |  |  |
| Patient variant at c.1739 | TTGGCCAAGTATTGGAAGCCT | GCTGGCTATCTGTTCCACTCA | This paper |
| RT-PCR |  |  |  |
| *ACTB*  Actin β | CTCCTTAATGTCACGCACGAT | CATGTACGTTGCTATCCAGGC | ^(28)^ |
| *PKG1*  Phosphoglycerate kinase1 | GTTGACCGAATCACCGACCT | GTCGACTCTCATAACGACCCG | ^(28)^ |
| *HPRT*  Hypoxanthine-guanine phosphor-ribo­syltransferase | ATGGACAGGACTGAACGTCT | TCCAGCAGGTCAGCAAAGAA | ^(28)^ |
| *GAPDH*  Glyceraldehyde-3 phosphate dehydrogenase | TCTTCTTTTGCGTCGCCAGCC | CCCAATACGACCAAATCCGTTGA | ^(28)^ |
| *LIG1* | TTTGTACGCCTTCGACCTCA | TGCTCGATGTCCTTGGTGTC | This paper |
| *LIG3 a+b* | CTTTTCAGCCAAGCCCAACA | CGAAACTCCCGTAGCAGACA | This paper |
| *LIG4* | CACCTTGCGTTTTCCACGAA | CAGATGCCTTCCCCCTAAGTTG | This paper |
| Ligation assay |  |  |  |
| Backbone | TAAGCGATGCTCTCACCGAGAATGGCAAGGGCCAGTTTTTCT | | ^(29)^ |
| nicked substrate | Phos- CTCGGTGAGAGCATCGCTTA | | ^(29)^ |
| nicked substrate with Dye | Dye-AGAAAAACTGGCCCTTGCCATT  Dye = dyomics781 | | This paper |

*Serum autoantibody microarray*

IgG and IgM autoantibodies testing were performed using a protein array platform at the Microarray Core Facility (University of Texas Southwestern Medical Center/UTSW, USA). In short, sera was incubated with self-antigens printed on a micro-chip, autoantibodies were detected with Cy3 and Cy5 anti-human IgG resp. IgM. Chips were read with a Genepix scanner, the resulting images were analyzed using Genepix Pro 6.0 software. For each antigen the net fluorescence intensity (NFI) was computed by subtracting the PBS control. The signal-to-noise ratio (SNR) was computed to decipher the true signal

$$\frac{{\mathrm{median}_{(Signal)}-median{}_{(Background)}}}{{{(SD}_{Background})}^{{}}}$$

Next an antibody-score was calculated = ln(NFI*SNR+1). R studio was used for the visualization and statistical computation - heatmaps were generated using the gplot heatmap.2 package, the Limma package^(11)^ was used to compute differential expression and empirical Bayes statistics.

*Generation of LIG4^–/–^ cells using CRISPR-Cas9*

Guide RNA (gRNA) sequence design was performed using open-source tools^(30, 31)^ for picking RNA guide sequences targeting exon 3 of *LIG4* with minimal identical additional genomic matches. Jurkat clone E6-1 (ATCC, cat# TIB-152^TM^) T cells were transfected with pSpCas9(BB)-2A-GFP (Addgene plasmid ID PX458), and either one gRNA or two gRNA targeting exon 3, using the Cell line Nucleofector^TM^ Kit V (Lonza, # VCA-1003). 24 hours later, into a 96-well plate and allowed to grow. Single-cell colonies were Sanger sequenced to confirm indels. Immunoblot and intracellular staining was utilized to confirm the absence of LIG4 protein in Jurkat LIG4^-/-^ cells.

| CRISPR Guides | Forward 5’-3’ | Reverse 5’-3’ | Source |
| --- | --- | --- | --- |
| sgRNA1 | CACCGCATCTCCATGAGTTCCAGT | AAACACTGGAACTCATGGAGATGC | This paper |
| sgRNA2 | CACCGAAAGAGAGAGAATGGCCTA | AAACTAGGCCATTCTCTCTCTTTC | This paper |
| Sanger | ATGGCTGCCTCACAAACTTC | TGCAACTCAGCAGCATCATT | This paper |

*Jurkat T cell LIG4 reconstitution assay*

A transient overexpression of LIG4 proteins utilizing a cytomegalovirus (CMV) promoter-driven *LIG4* expression vector pRP[Exp]-mCherry/Puro-CAG>hLIG4[NM_001352604.2] (Vector Builder) was performed in the obtained *LIG4*-KO Jurkats. To generate R580Q and A842D mutant LIG4-expressing vectors, the plasmid was subjected to KOD-plus-mediated site-directed mutagenesis (Toyobo) and isolated for single colonies with the following primers.

| Mutagenesis | Forward 5’-3’ | Reverse 5’-3’ | Source |
| --- | --- | --- | --- |
| R580Q | CTGCACCTTGCGTTTTCCACAAATTGAAAAGATAAGAGATGA | TCATCTCTTATCTTTTCAATTTGTGGAAAACGCAAGGTGCAG | This paper |
| A842D | GGACAAGGTTAGCTATTAAAGACTTGGAGCTTCGGTT | AACCGAAGCTCCAAGTCTTTAATAGCTAACCTTGTCC | This paper |

Designed plasmids were purified with EndoFree Maxi plasmid purification kit (Qiagen) and sanger-sequenced for the full *LIG4* region to confirm mutagenesis specificity. Endogenous and transfection-derived expression of LIG4 proteins were confirmed by two-step intracellular staining using Cytofix/Cytoperm buffer (BD), rabbit monoclonal anti-human LIG4 (Clone JM64-32, Thermo Scientific) and mouse anti-rabbit IgG-Alexa Flour 647 (Cell Signaling Technologies).

Generated *LIG4*-KO Jurkats, cultured in RPMI medium supplemented with 10% fetal bovine serum (FBS) and penicillin/streptomycin (P/S) (R10+), were seeded (100μl) in quadruplicate in 96-well U-bottomed plates (Falcon). LIG4-expression plasmid DNAs of indicated mixture ratios were incubated with Xfect Transfection Reagent buffer/polymer (Takara Clontech). The mixture was then incubated with CombiMag (OZ Biosciences) and the obtained reaction was applied to each well and subsequently subjected to magnetofection with a 96-well plate-size magnetic plate (OZ Biosciences) and incubated for 20 hours at 37˚C, 5% CO2. Next, 100 μl of R10+ was added to each well with or without bleomycin added at a final concentration of 5 μM. Cells were incubated for 9 hours and subsequently stained with Annexin V-APC (Biolegend) in a Ca2+-positive buffer (Biolegend). Stained cells were analyzed on a 4-laser (Violet/Blue/Yellow-green/Red) FACS LSRII Fortessa (BD). Approximately 30,000 cells were acquired for each sample. Results were analyzed via FACS Diva (BD) and FlowJo ver 9 (BD). The percentage of cell death prevention was calculated as 100 x {1-(∆% Annexin V+ frequencies in mCherry-positive Jurkats)/(∆% Annexin V+ frequencies in mCherry-negative Jurkats)} (representative calculation shown in FIG. 7D), with ∆% obtained by subtraction from the triplicate or quadruplicate mean of each value in bleomycin-unstimulated wells. Negative values in the parentheses were substituted as zero, which did not appear in the mCherry marker-negative denominator. Calculated assays were performed twice or three times in triplicate or quadruplicate, and pooled for ANOVA analysis.

*Statistical analysis*

For statistical evaluation, the tests that were utilized are specified in each figure legend. Error bars show the standard deviation (SD) centered on the mean unless otherwise indicated. P values were compared to an α-threshold of 0.05, set as an arbitrary significance level. Data were analyzed with GraphPad Prism software (version 7-9) and a linear model for differential expression analysis was performed using the R platform. Specific tests are detailed in the figure legends.

**Table EI| Hematological and immunological parameters in patients with heterozygous LIG4 variants.**

|  |  | | |  |  | | Affected subjects | | | |
| --- | --- | --- | --- | --- | --- | --- | --- | --- | --- | --- |
|  |  | | |  |  | | P1 R580Q | P2 Father R580Q | P3 A842D | P4 A842D |
|  |  | | | Gender, Age | | F 21y | | M 50y | F 31y | M 51y |
| Clinical Phenotype |  | | |  |  | |  |  |  |  |
| Susceptibility to Infection  Autoimmunity  Autoinflammation  Lymphoproliferation | |  |  | |  | | Yes (Figure 1)  Yes (Figure 1)  Recurrent Fever  Splenomegaly | Mild  Cytopenias  No  ND | Recurrent Pneumonias  No  Colitis  Splenomegaly | Airway infections  Thyreoiditis  Colitis  No |
| Hemoglobin | g/L | | | (m 140-180, f 120-160) |  | | 113 🡫 | 140 | 149 | 151 |
| MCV | fl | | | (81-100) |  | | 80.5 | 83.3 | 87 | 82 |
| Thrombocytes | x10^9^/L | | | 150-450 |  | | 116 🡫 | 159 | 246 | 123 🡫 |
| WBC | Leukocytes | | | (3.5-10 x10^9^/L) |  | | 3.22 🡫 | 5.23 | 10.89 🡩 | 4.74 |
|  | Neutrophils | | | 40-74% (1.3-6.7 x10^9^/L) |  | | 75.2% (2.42) | 65.1% (3.47) | 75.4% (8.20) | 72.1% (3.42) |
|  | Monocytes | | | 3.4-9% (0.12- 0.62 x10^9^/L) |  | | 6.5% (0.21) | 8.9% (0.47) | 5.6% (0.61) | 4.8% (0.23) |
|  | Eosinophils | | | 0-7% (0-0.3 x10^9^/L)) |  | | 1.2% (0.04) | 4.3% (0.23) | 0.6% (0.07) | 2.2% (0.10) |
|  | Basophils | | | 0-1.5% (0-0.09 x10^9^/L) |  | | 0.9% (0.03) | 0.8% (0.04) | 0.6% (0.07) | 0.4% (0.02) |
|  | Total lymphocytes | | | 20-45% (0.9-3.3 x10^9^/L) |  | | 15.2% (0.5) 🡫 | 19.2% (1.0) | 17.8% (1.9) 🡫 | 19.1% (0.9) 🡫 |
| % of lymphocytes | Total T cells, CD3^+^ | | | 55-86%  [742-2750 /uL] |  | | 75% | 76% | 76% | 65% 🡫 |
|  |  |  |  |  |  |  | [841/uL] |  | [1593/uL] | [651/uL] 🡫 |
|  | Total B cells, CD19^+^CD20^+^ | | | 5-22%  [80-616 /uL] |  | | 15%  [171/uL] | 7.8% | 17%  [354/uL] | 27% [274/uL] 🡩 |
|  | Total NK cells, CD56^+^CD16^+^ | | | 5-26%  [84-724 /uL] |  | | 9%  [99/uL] |  | 7%  [140/uL] | 6% 🡫  [62/uL] 🡫 |
|  | Surface markers | | |  | | | | | | |
| Total helper T | CD3^+^ CD4^+^ | | | 33-58% [404-1612/uL] |  | | 61% 🡩 | 63.5% 🡩 | 37% | 67% 🡩 |
|  |  |  |  |  |  |  | [685/uL] |  | [793/uL] | [371/uL] 🡫 |
| Helper T, naïve | CD27^+^ CD45RO^-^ | | | 15.7-54.7% |  | | 0.5% 🡫 | 9.75% 🡫 | 5.9% 🡫 | 13.3% 🡫 |
| Helper T, central memory | CD27^+^ CD45RO^+^ | | | 8-28.8% |  | | 7.6% 🡫 | 73.8% 🡩 | 32.1% 🡩 | 34.2 🡩 |
| Helper T, effector memory | CD27^-^ CD45RO^+^ | | | 16.8-57.4% |  | | 87.3% 🡩 | 15.1% 🡫 | 60.5% 🡩 | 49.6% |
| Helper T, follicular | CXCR5^+^PD1^+^ | | | 6.9-19.1% |  | | 39.8% 🡩 |  | 30.4% 🡩 | 28.3% 🡩 |
| Helper T, regulatory | CD25^hi^CD127^low^ | | | 6.1-11% |  | | 2.3% 🡫 | 3.41% 🡫 | 5% 🡫 | 10.8% |
| Helper, recent thymic emigrants | CD31^+^CD27^+^CD45R0^-^ | | | 14.1-37.2% (% of CD4+) |  | | 1% 🡫 |  | 6.4% 🡫 | 12.6% 🡫 |
| Total cytotoxic T | CD3^+^ CD8^+^ | | | 13-39% [220-1129/uL] |  | | 14% | 32.2% | 36% | 21% |
|  |  |  |  |  |  |  | [163/uL] 🡫 |  | [753/uL] | [176/uL] 🡫 |
| Cytotoxic T, naïve | CD27^+^ CD45RO^-^ | | | 7-62.5% |  | | 2% 🡫 | 12.7% | 3.5% 🡫 | 17.0% |
| Cytotoxic T, central memory | CD27^+^ CD45RO^+^ | | | 0.6-4.4% |  | | 0.4% 🡫 | 62.2% 🡩 | 1.6% | 7.1% 🡩 |
| Cytotoxic T, effector memory | CD27^-^ CD45RO^+^ | | | 4.3-64.5% |  | | 68.3% 🡩 | 17.2% | 71.8% 🡩 | 57.3% |
| Cytotoxic, TEMRA | CD45RA^+^CD62L^-^ | | | 8.1-60.5% |  | | 29.2% |  | 23.1% | 18.6% |
| Total double negative T | TCRαβ^+^TCRγδ^-^CD8^-^CD4^-^ | | | ≤ 2.5% of (of CD3^+^) |  | | 0.66% | 2.68% 🡩 | 3.2% 🡩 | ND |
| Total, γδ T | TCRβ^-^TCRγδ^+^ | | |  |  | | 3.21% | 2.45% | ND | ND |
| Total, Vα7.2^+^ | Vα7.2^+^ CD3^+^ | | | Mean 5.82% (of CD3^+^) |  | | 1% | 2.94% | 1.84% | 1.87% |
| Total, MAIT | Vα7.2^+^ CD161^+^CD3^+^ | | | 1.55-6.77% (of CD3^+^) |  | | 0.47% | 1.41% | ND |  |
| Plasmablasts | CD27^+^CD38^+^CD20^-^CD19^+^ | | | 0.1-3% (of CD19^+^ cells)  [1-5/uL] |  | | <0.1% 🡫  [<1/uL] 🡫 | 0.24% | <0.1% 🡫  [<1/uL] 🡫 | 1.9%  [4/uL] |
| Naïve B | IgD^+^IgM^+^CD27^-^ | | | 25.1-92.4% [66-228/uL] |  | | 60.5% [81/uL] | 30.6% | 60.6% [215/uL] | 93.3%  [209/uL] |
| Switched memory B | IgD^-^IgM^-^CD27^+^ | | | 2.4-32.6% [8-102/uL] |  | | 0.5% [<1/uL] 🡫 | 15% | 4% [14/uL] | 2.5% [4/uL] 🡫 |
| Transitional B | IgD^+^IgM^+^CD38^+^ | | | 0.3-2.9% [1-5/uL] |  | | 0.4% [<1/uL] 🡫 | 0.56% | 0.1% [<1/uL] 🡫 | 3.2% [7/uL] 🡩 |
| Marginal zone like B | IgD^+^IgM^+^CD27^+^ | | | 3.1-59.7% [8-172/uL] |  | | 32.3% [43/uL] | 26% | 28.7% [102/uL] | 0.7% 🡫  [2/uL] 🡫 |
| CD21^low^ B cells | CD21^low^ | | | 0.5-4.7% [1-12/uL] |  | | 6.8% [9/uL] 🡩 | 7.2% 🡩 | 2.3% [8/uL] | 0.9% [2/uL] |
| Immunoglobulins |  | | |  | | | | | | |
| IgG |  | | | 7-16 g/L |  | | <0.1 🡫 | 6.58 🡫 | 2.8 🡫 | 4.9 🡫 |
| IgM |  | | | 0.4-2.3 g/L |  | | 0.25 🡫 | 1.32 | <0.05 🡫 | 0.34 🡫 |
| IgA |  | | | 0.7-4 g/L |  | | < 0.06 🡫 | 0.58 🡫 | <0.05 🡫 | 0.69 🡫 |

F female, Ig immunoglobulin, MAIT mucosa associated invariant T cell, m male, MCV mean corpuscular volume, ND not determined, NK Natural killer, WBC white blood count. Red shading indicates values higher than the in-house reference range; blue shading indicates the value is below the in-house reference range. The majority of immune cell subsets listed in this table were temporarily linked with the immunoglobulin quantification shown here. 🡩/🡫: Above/below reference range.

**Table EII|** Whole-exome sequencing information regarding the missense variant in LIG4 and FAS.

| Genetic location | Nucleotide change  (c.DNA) | Zygosity | Gene symbol | AA change | rsID | ESP | Poly  Phen2 | SIFT | CADD-PHRED | gnomAD  allele freq v2.1.1 | gnomAD  allele freq v3.1.2 |
| --- | --- | --- | --- | --- | --- | --- | --- | --- | --- | --- | --- |
| 13_108209530 | c.A1739G | Het | *LIG4* | p.R580Q  R[C**G**A] > Q[C**A**A] | rs14661  6552 | 0.0 | Deleter­ious, 1 | Deleter­ious, 0 | 33 | 8.4^-5^ | 3.6^-4^ |
| 10_90768694 | c.G383A | Homo (Father)  Het (P1, Brother) | *FAS* | p.R128K  R[AGA] >  K[AAA] | na | 0.0 | 0.541 | 0.241 | 23.6 | na | na |
| 13-108208744 | c.C2525A | Het | *LIG4* | p.A842D  A[GCC] > D[GAC] | rs7266 0870 |  | 0.425 | Deleter­ious | 23 | 1.11^-3^ | 1.26^-3^ |

AA amino acid, Allele freq allele frequency, CADD-PHRED Combined Annotation Dependent Depletion, Chr. chromosome, ESP Exome Sequencing Project, freq. frequency, gnomAD Genome Aggregation Database [accessed 01.2023], Het heterozygous, PolyPhen2 Polymorphism Phenotyping, rsID single nucleotide polymorphism database identifier, SIFT Sorting for Intolerant from Tolerant.

**Supplemental References**

1. Navarini AA, Hruz P, Berger CT, Hou TZ, Schwab C, Gabrysch A, et al. Vedolizumab as a successful treatment of CTLA-4-associated autoimmune enterocolitis. J Allergy Clin Immunol. 2017;139(3):1043-6 e5.

2. Burgener AV, Bantug GR, Meyer BJ, Higgins R, Ghosh A, Bignucolo O, et al. SDHA gain-of-function engages inflammatory mitochondrial retrograde signaling via KEAP1-Nrf2. Nat Immunol. 2019;20(10):1311-21.

3. McKenna A, Hanna M, Banks E, Sivachenko A, Cibulskis K, Kernytsky A, et al. The Genome Analysis Toolkit: a MapReduce framework for analyzing next-generation DNA sequencing data. Genome Res. 2010;20(9):1297-303.

4. Ghraichy M, Galson JD, Kovaltsuk A, von Niederhausern V, Pachlopnik Schmid J, Recher M, et al. Maturation of the Human Immunoglobulin Heavy Chain Repertoire With Age. Front Immunol. 2020;11:1734.

5. Vander Heiden JA, Yaari G, Uduman M, Stern JN, O'Connor KC, Hafler DA, et al. pRESTO: a toolkit for processing high-throughput sequencing raw reads of lymphocyte receptor repertoires. Bioinformatics. 2014;30(13):1930-2.

6. Gupta NT, Vander Heiden JA, Uduman M, Gadala-Maria D, Yaari G, Kleinstein SH. Change-O: a toolkit for analyzing large-scale B cell immunoglobulin repertoire sequencing data. Bioinformatics. 2015;31(20):3356-8.

7. Ye J, Ma N, Madden TL, Ostell JM. IgBLAST: an immunoglobulin variable domain sequence analysis tool. Nucleic Acids Res. 2013;41(Web Server issue):W34-40.

8. Lunter G, Goodson M. Stampy: a statistical algorithm for sensitive and fast mapping of Illumina sequence reads. Genome Res. 2011;21(6):936-9.

9. Yaari G, Uduman M, Kleinstein SH. Quantifying selection in high-throughput Immunoglobulin sequencing data sets. Nucleic Acids Res. 2012;40(17):e134.

10. Daley SR, Koay HF, Dobbs K, Bosticardo M, Wirasinha RC, Pala F, et al. Cysteine and hydrophobic residues in CDR3 serve as distinct T-cell self-reactivity indices. J Allergy Clin Immunol. 2019;144(1):333-6.

11. Ritchie ME, Phipson B, Wu D, Hu Y, Law CW, Shi W, et al. limma powers differential expression analyses for RNA-sequencing and microarray studies. Nucleic Acids Res. 2015;43(7):e47.

12. Bienert S, Waterhouse A, de Beer TA, Tauriello G, Studer G, Bordoli L, et al. The SWISS-MODEL Repository-new features and functionality. Nucleic Acids Res. 2017;45(D1):D313-D9.

13. Humphrey W, Dalke A, Schulten K. VMD: Visual molecular dynamics. Journal of Molecular Graphics. 1996;14(1):33-8.

14. Pettersen EF, Goddard TD, Huang CC, Couch GS, Greenblatt DM, Meng EC, et al. UCSF Chimera--a visualization system for exploratory research and analysis. J Comput Chem. 2004;25(13):1605-12.

15. Bignucolo O, Berneche S. The Voltage-Dependent Deactivation of the KvAP Channel Involves the Breakage of Its S4 Helix. Front Mol Biosci. 2020;7:162.

16. Vanommeslaeghe K, Hatcher E, Acharya C, Kundu S, Zhong S, Shim J, et al. CHARMM general force field: A force field for drug-like molecules compatible with the CHARMM all-atom additive biological force fields. J Comput Chem. 2010;31(4):671-90.

17. Irwin JJ, Sterling T, Mysinger MM, Bolstad ES, Coleman RG. ZINC: a free tool to discover chemistry for biology. J Chem Inf Model. 2012;52(7):1757-68.

18. Jorgensen WL, Chandrasekhar J, Madura JD, Impey RW, Klein ML. Comparison of simple potential functions for simulating liquid water. The Journal of Chemical Physics. 1983;79(2):926-35.

19. Kaminski AM, Tumbale PP, Schellenberg MJ, Williams RS, Williams JG, Kunkel TA, et al. Structures of DNA-bound human ligase IV catalytic core reveal insights into substrate binding and catalysis. Nat Commun. 2018;9(1):2642.

20. Van Der Spoel D, Lindahl E, Hess B, Groenhof G, Mark AE, Berendsen HJ. GROMACS: fast, flexible, and free. J Comput Chem. 2005;26(16):1701-18.

21. MacKerell AD, Bashford D, Bellott M, Dunbrack RL, Evanseck JD, Field MJ, et al. All-atom empirical potential for molecular modeling and dynamics studies of proteins. J Phys Chem B. 1998;102(18):3586-616.

22. Hess B, Bekker H, Berendsen HJC, Fraaije JGEM. LINCS: A linear constraint solver for molecular simulations. Journal of Computational Chemistry. 1997;18(12):1463-72.

23. Essmann U, Perera L, Berkowitz ML, Darden T, Lee H, Pedersen LG. A smooth particle mesh Ewald method. The Journal of Chemical Physics. 1995;103(19):8577-93.

24. Jo S, Lim JB, Klauda JB, Im W. CHARMM-GUI Membrane Builder for mixed bilayers and its application to yeast membranes. Biophys J. 2009;97(1):50-8.

25. Baker NA, Sept D, Joseph S, Holst MJ, McCammon JA. Electrostatics of nanosystems: application to microtubules and the ribosome. Proc Natl Acad Sci U S A. 2001;98(18):10037-41.

26. Kumari R, Kumar R, Open Source Drug Discovery C, Lynn A. g_mmpbsa--a GROMACS tool for high-throughput MM-PBSA calculations. J Chem Inf Model. 2014;54(7):1951-62.

27. Pfaffl MW. A new mathematical model for relative quantification in real-time RT-PCR. Nucleic Acids Res. 2001;29(9):e45.

28. Bigler MB, Egli SB, Hysek CM, Hoenger G, Schmied L, Baldin FS, et al. Stress-Induced In Vivo Recruitment of Human Cytotoxic Natural Killer Cells Favors Subsets with Distinct Receptor Profiles and Associates with Increased Epinephrine Levels. PLoS One. 2015;10(12):e0145635.

29. Conlin MP, Reid DA, Small GW, Chang HH, Watanabe G, Lieber MR, et al. DNA Ligase IV Guides End-Processing Choice during Nonhomologous End Joining. Cell Rep. 2017;20(12):2810-9.

30. Ran FA, Hsu PD, Wright J, Agarwala V, Scott DA, Zhang F. Genome engineering using the CRISPR-Cas9 system. Nat Protoc. 2013;8(11):2281-308.

31. Labun K, Montague TG, Krause M, Torres Cleuren YN, Tjeldnes H, Valen E. CHOPCHOP v3: expanding the CRISPR web toolbox beyond genome editing. Nucleic Acids Res. 2019;47(W1):W171-W4.

32. Frey-Jakobs S, Hartberger JM, Fliegauf M, Bossen C, Wehmeyer ML, Neubauer JC, et al. ZNF341 controls STAT3 expression and thereby immunocompetence. Sci Immunol. 2018;3(24).

33. Oliveira JB, Bleesing JJ, Dianzani U, Fleisher TA, Jaffe ES, Lenardo MJ, et al. Revised diagnostic criteria and classification for the autoimmune lymphoproliferative syndrome (ALPS): report from the 2009 NIH International Workshop. Blood. 2010;116(14):e35-40.

34. Jumper J, Evans R, Pritzel A, Green T, Figurnov M, Ronneberger O, et al. Highly accurate protein structure prediction with AlphaFold. Nature. 2021;596(7873):583-9.

**Figure legends Supplemental Figures and Video**

**FIG E1| Qualitative changes in T_regs_ and dysregulated B cell tolerance in P1 and her father. A)** Circulating CD4^+^CD25^hi^ CD127^low^ T regulatory cells (T_reg_) evaluation. **B)** Surface molecules expression within T_reg_ cells using flow cytometry. **C)** CD4^+^ T helper subsets^(32)^ characterization. T_H1_ (CCR6^-^CD45RA^-^CXCR3^+^CCR4^-^), T_H1_* (CCR6^+^CD45RA^-^CXCR3^+^CCR4^-^), T_H2_ (CCR6^-^CD45RA^-^CXCR3^-^CCR4^+^) and T_H17_ (CCR6^+^CD45RA^-^CXCR3^-^CCR4^+^). **D)** Volcano plot displaying the father’s serum autoantibody IgG - detected by protein microarray, and compared to HD serum. Red: auto-antigens which were significantly more recognized by the father’s IgGs. **E)** The autoantibody score for the father’s IgGs, which were significantly higher compared to HDs. **F)** Heatmap with hierarchical clustering of all autoantibodies specificities. Antigens detected with a significant positive fold change by the father’s serum IgGs are marked with arrow heads on the top. (A) Kruskal-Wallis test with Dunn’s correction, (B/C) Mann-Whitney test with multiple comparison correction. (D/E) fold change calculation by fitting a linear model/empirical Bayes statistics. * p < 0.05.

**FIG E2| P1’s and her father’s peripheral lymphocytes demonstrate preserved B and T cell receptor repertoires. A)** High throughput sequencing experiment of the T cell receptor β-chain (*TRB*) loci of peripheral blood-derived bulk T cells. The most variable non-germline region of the TCR, namely the (complementary determining region 3) CDR3 was investigated regarding the relative distribution of its length within unique *TCRB* sequences. The CDR3 length distribution of the healthy mother is highlighted as a grey shadow. **B)** For the analysis of *VJ* gene pairing within total *TCRA,* heatmaps were computed displaying the *V*-gene (rows) and *J*-gene (columns) pairing frequency. Most distal *V* with distal *J* gene pairing is indicated by a box in the top right. **C)** Peripheral blood B cells were subjected to high-throughput RNA sequencing of the *IGH* locus. Transcripts of total sequences (seq.) were used to determine the constant region utilization (naïve, MD memory, IgG, IgA) in the two patients and three healthy donors.

**FIG E3 | Characterisation of a novel *FAS* variant*.* A)** Representative flow-cytometry plots of naïve CD4^+^ T cell subpopulation (CD27^+^CD45RO^-^, gray shading). **B** and **C)** Representative flow-cytometric investigation of double-negative (DNT) T cells (gray box) and it’s frequency within the TCRαβ^+^TCRγδ^-^ T cells. Gray shading indicates the autoimmune lymphoproliferative syndrome (ALPS)-related threshold for DNTs^(33)^. **D)** Serum concentrations of vitamin B12 (Vit B12, left side) and soluble FAS ligand (sFasL, right side) were measured at multiple time points in P1. Gray shading indicates the ALPS-related diagnostic thresholds^(33)^. **E)** *In vitro* apoptosis assay using phytohemagglutinin (PHA)-induced T cell blasts. Annexin V binding was measured by flow cytometry after four hours of stimulation with either anti-CD3 (OKT3), anti-FAS, recombinant FAS-ligand (rec FASL) and staurosporine (Stauro). **F)** So far, there is no solved FAS structures, bearing the region of interest. Consequently, we generated protein using the Alphafold^(34)^ algorithm, model superposition was achieved using VMD^(13)^. The algorithm hardly identified an effect of the variant on the protein backbone. The magnified view shows that two acidic residues, both located on an adjacent b-strand, may sandwich residue 128, likely maintaining its sidechain in an extended position toward the protein exterior.

**FIG E4 |*In silico* decreased DNA binding capacity of the mutant LIG4 R580Q. A)** Molecular dynamic simulations were used to quantify the total binding energy of the enzyme and the DNA. In each panel, the results from six independent trajectories (>500ns) for the WT and R580Q protein are shown. Right side: average for each time series analysis is depicted. Left side: average of all trajectories per enzyme. **B)** The binding energy difference (mutant – WT) was investigated as a function of the residue position (x-axis). **C)** Representative time-series of the torsion angle f for the WT *vs.* R580Q residue side-chains show that Gln580 is less stable than Arg580. **D)** Distribution of the WT *vs*. R580Q residue side-chains φ angles. The dihedrals were extracted at an interval of 1ns over the last 100ns of simulation. **E)** Representative time-series analysis regarding the torsion angle ψ for the WT *vs*. R580Q residue side-chains. **F)** Distribution of the WT *vs*. R580Q residue side-chains ψ angles. The dihedrals were extracted at intervals of 1ns over the last 100ns of simulation. Quantification of the number of **G)** salt bridges (for the WT) *vs.* **H)** hydrogen bonds (for the R580Q) formed over time between the residue and the DNA backbone. Significance testing in (a) was performed using the Mann-Whitney test. Mean ± SEM.

**FIG E5 | Domino skewing of LIG4-XRCC4 salt bridge interactions upon LIG4 A842D substitution in MD simulation analysis**.

Averaged distance values during quadruplicate independent trajectories for indicated residues are compared and analyzed by Kruskal-Wallis H test (p < 0.05: single-star). These describe a domino-effect of the A842D mutation involving four pairs of acidic and basic residues located in LIG4 BRCT2 and XRCC4 (FIG 6, *D*). First, in wild type (WT) LIG4, Glu170 of XRCC4 builds a strong salt bridge with Arg846 of LIG4 BRCT2, with a distance between the carboxylic atoms and the guanidium group varying among the four replications between 2.7 and 2.9Å. In the LIG4 A842D variant, this salt bridge is disrupted in two of the trajectories (comparison 1). In WT, Glu170 is the only acidic residue located within a range allowing the formation of a salt bridge whereas in the A842D variant Arg846 builds a strong interaction with the neighboring carboxyl group of Asp842; i.e., in the variant Arg846 can exchange interactions either with Glu170 in XRCC4 or Asp842 in BRCT2. Further, the interaction with Asp842 reorients Arg846 in an orientation favorable to build an additional salt bridge with Glu163 of XRCC4 (comparison 2). This salt bridge is absent in the WT, where Glu163 interacts transiently with Lys164 of the same chain. As expected, this interaction is slightly weakened in the variant: the interaction between Glu163 and Arg846 disfavors the interaction between Glu163 and Lys164 (comparison 3). A more distant residue affected by the mutation in these simulations is Lys832 which, attracted by the negatively charged Asp842, reorients and consequently approaches Arg846 (comparison 4). However, the large distance between Glu163 and Lys832 corresponds to a very weak interaction, not affected by the mutation. Further salt bridges, like for example the one built by Asp157 and Arg161 on XRCC4 B (comparison 5), are not affected by the mutation in these simulations.

In summary, the interaction between Asp842 and Arg846 displaces the interactions of LIG4 BRCT2 with XRCC4, inducing a salt bridge with Glu163, and weakening the salt bridge initially formed with Glu170. This could lead to a tension or a shift of the binding along the XRCC4 helices. This salt bridge network modification further augments conformational freedom of Lys164 on the opposite side of the XRCC4 helix.

**FIG E6 | CD4+ T-cell subset frequency linkage with LIG4 mutation-associated DNA damage-driven cell death.** A) Left: CD4+ T-cell subset-level dead cell frequencies following 24-hour bleomycin exposure in Index patients P1, P3 and P4 aligned with healthy (n = 13) and disease (FG375/FG278/FG169) controls. Performed in pooled triplicate/quadruplicate (P1), duplicate/triplicate (P3) or triplicate/quadruplicate (HCs/DCs/P4). Naïve CD4-depleted FG169 is shown in star icon. Right: Post-hoc p values of one-way ANOVA analysis for bleomycin-treated groups. B) Representative flow cytometric plots of CD45RO-CD27 gating in CD4+ T-cell populations in P1, P3 and P4. Anti-CD27 conjugated with Brilliant Violet 650 in P3 and FITC in P1/P4. C) CD45RO-CD27+ naïve (top) and CD45RO+CD27+ central memory (bottom) CD4+ T-cell frequencies of healthy controls (gray), disease controls (green) and monoallelic LIG4-mutated patients (pink). Value for P2 adopted from Fig. 1. Values for P1 and P4 are represented as average of triplicate, hence not exactly matching values in flow plot. D) Comparison of naïve and central memory CD4+ T-cell frequencies in healthy controls versus patients P1, P3 and P4 by unpaired t tests.

**Video E1 | Weaker interaction of the residue R580Q with the DNA as compared to WT**. The video shows a trajectory of the WT, left, and R580Q mutant, right, and their respective interaction with the DNA duplex interaction. The respective residues 580, are depicted as sticks with the carbon, nitrogen respectively, oxygen atoms colored in black, blue and red. Spheres appear when the residue side-chain is <4Å of oxygen (red) or phosphate atoms (brown) of the adenylated DNA strand.

Link: https://www.dropbox.com/s/08vqclai7n7z5iq/lig4.wt1.r580q1.210125.mov?dl=0
