# Supplementary figures and images for: Autoimmunity and immunodeficiency associated with monoallelic LIG4 mutations via haploinsufficiency
Source: J Allergy Clin Immunol. Author manuscript; Available in PMC 2024 Feb 1. (PMC10529397; doi:10.1016/j.jaci.2023.03.022)

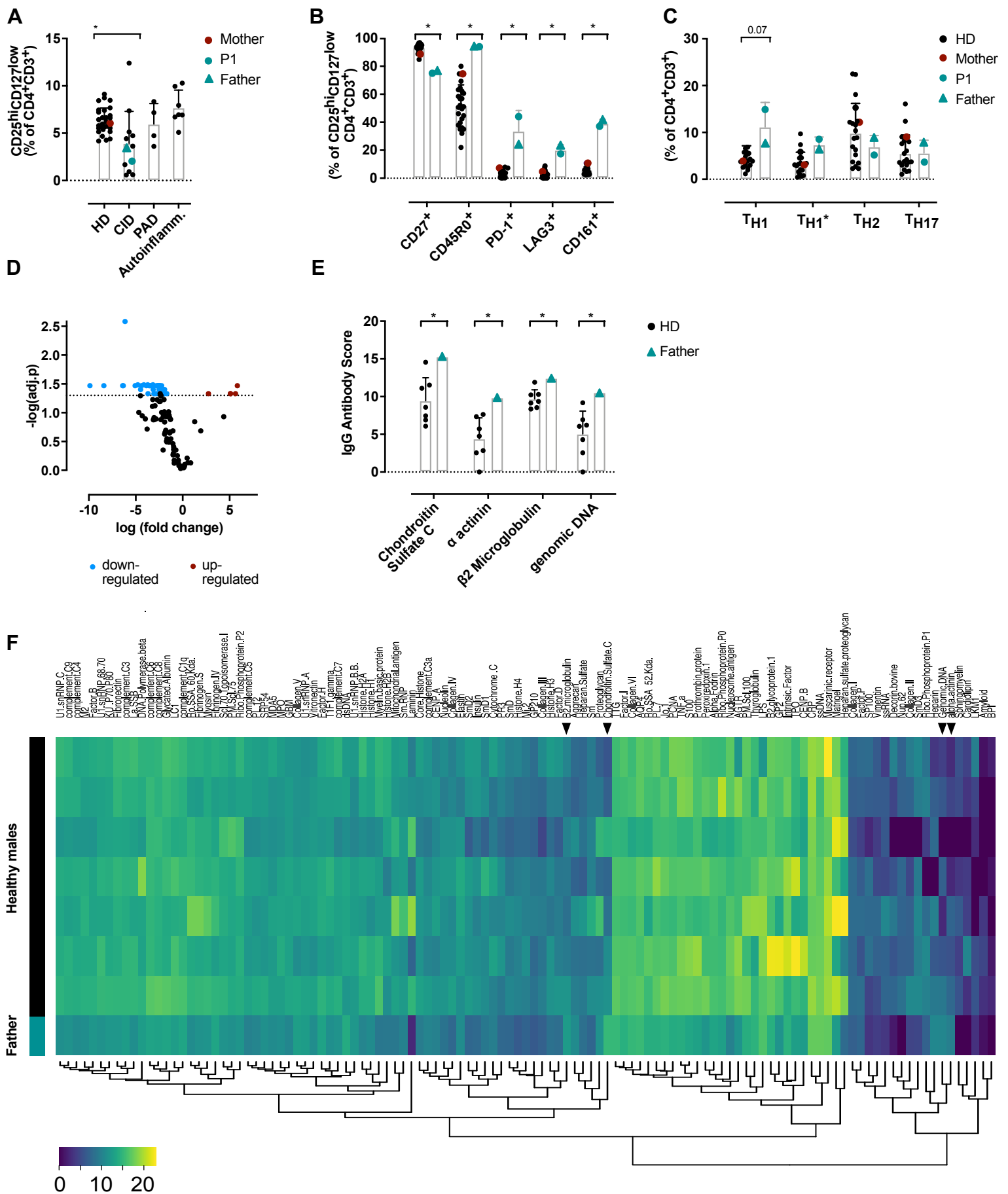

Supplement: Supplemental Figure E1 [file NIHMS1915164-supplement-Supplemental_Figure_E1.pdf]

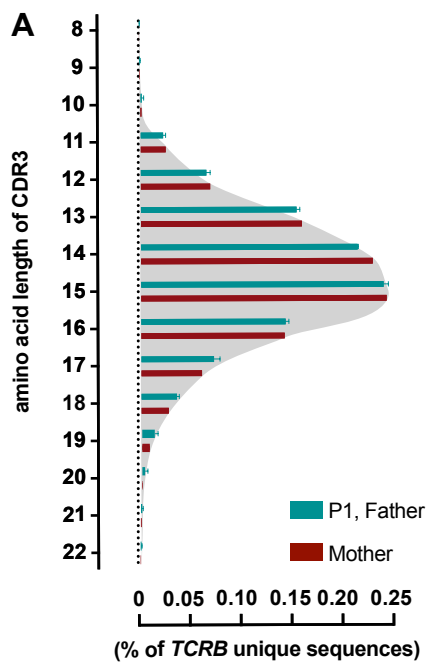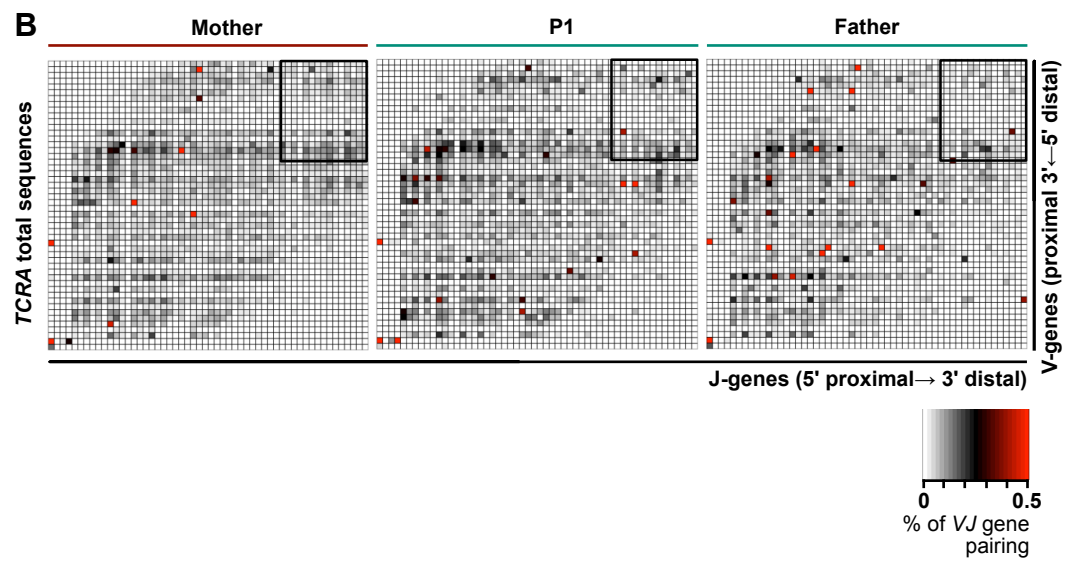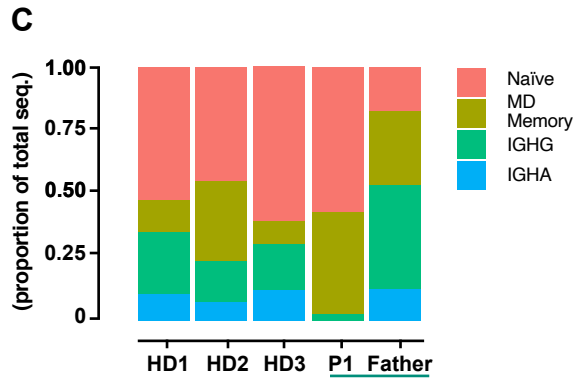

Supplement: Supplemental Figure E2 [file NIHMS1915164-supplement-Supplemental_Figure_E2.pdf]

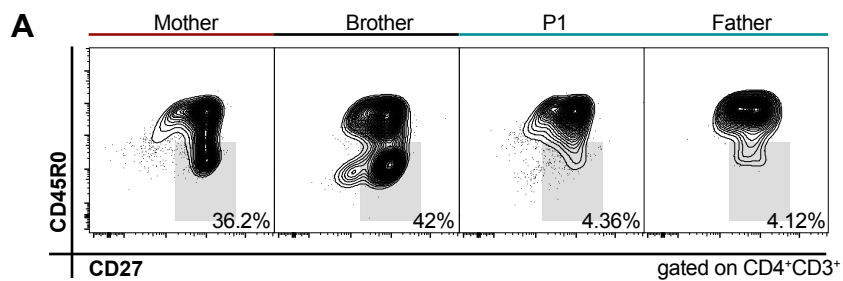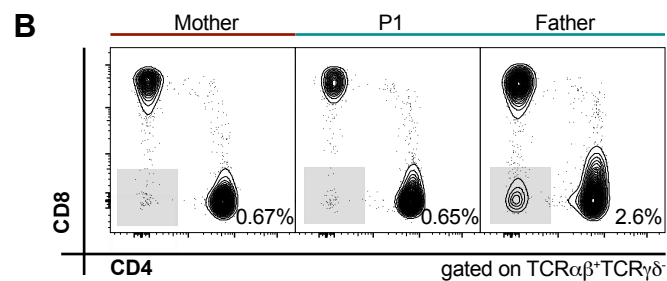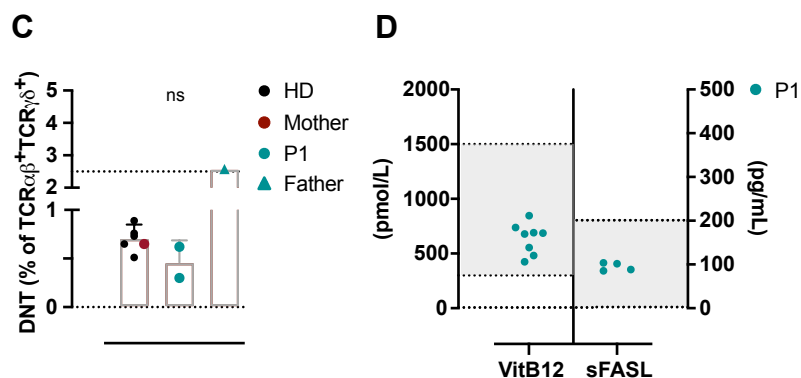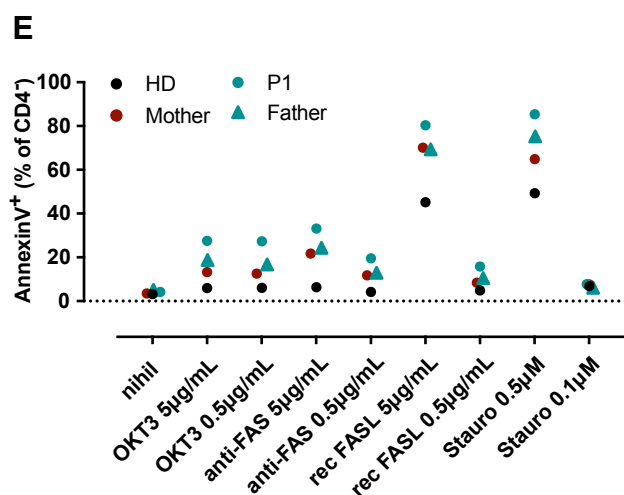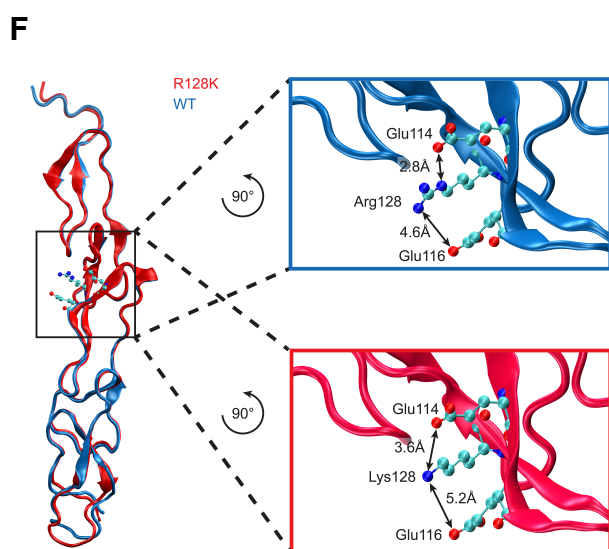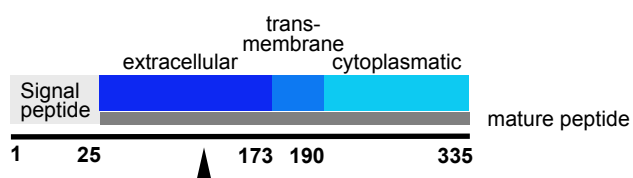

Supplement: Supplemental Figure E3 [file NIHMS1915164-supplement-Supplemental_Figure_E3.pdf]

A

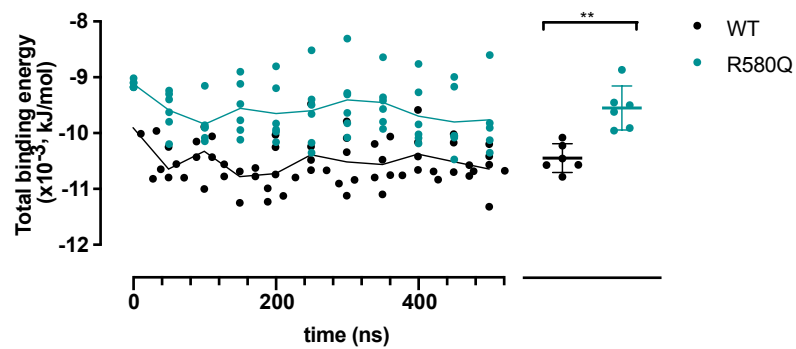

B

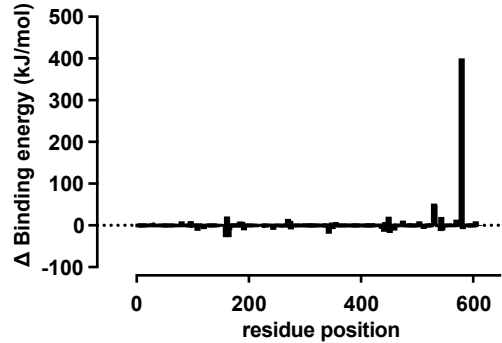

C

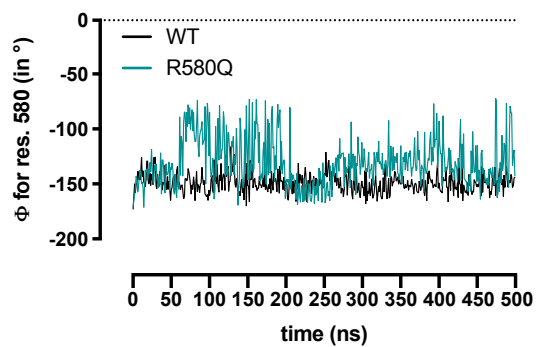

D

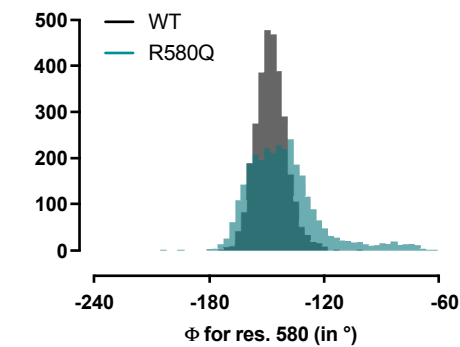

E

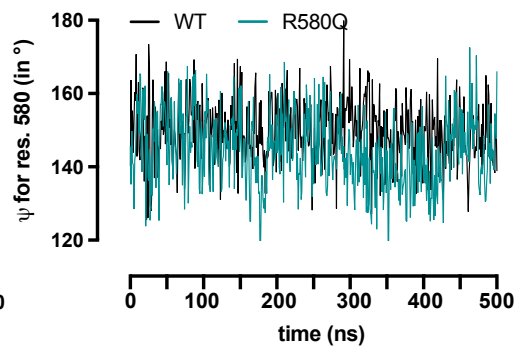

F

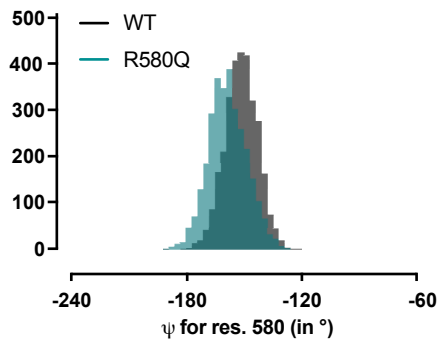

G

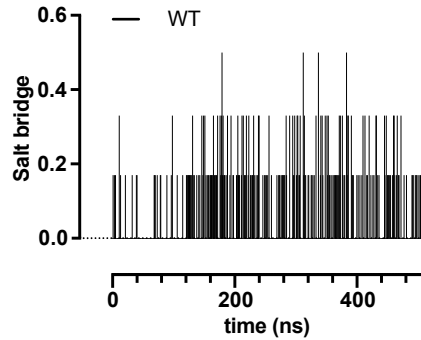

H

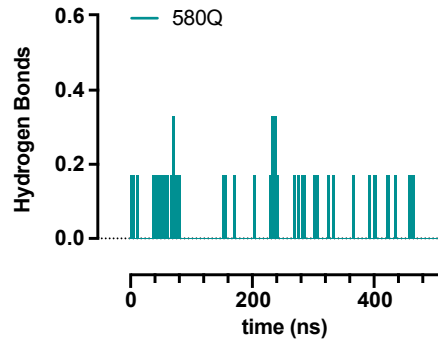

Supplement: Supplemental Figure E4 [file NIHMS1915164-supplement-Supplemental_Figure_E4.pdf]

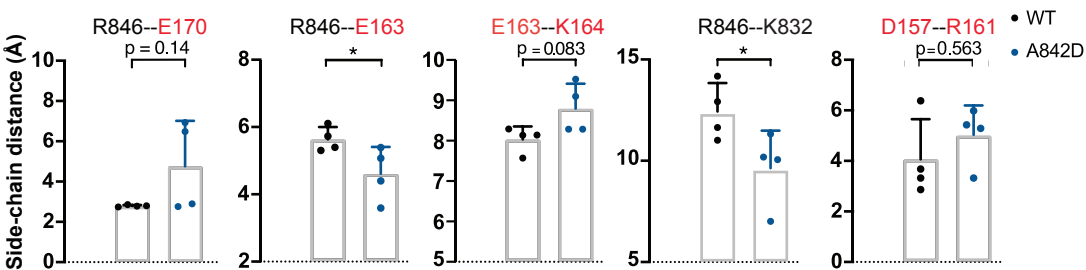

Supplement: Supplemental Figure E5 [file NIHMS1915164-supplement-Supplemental_Figure_E5.pdf]

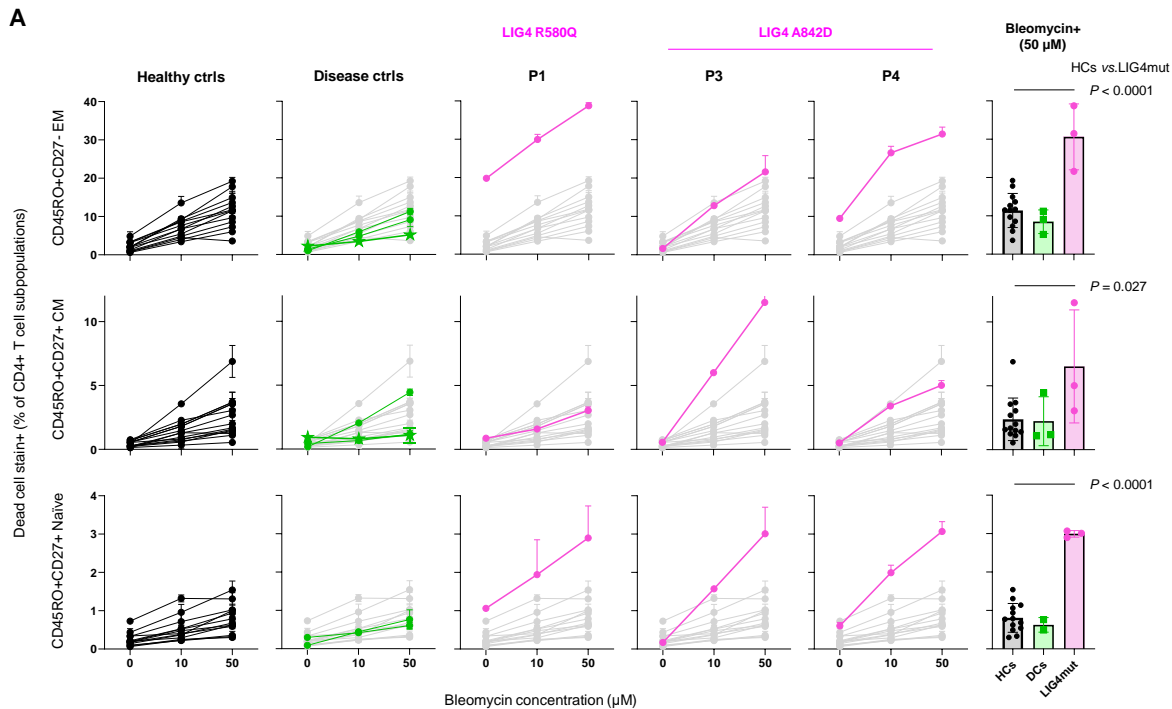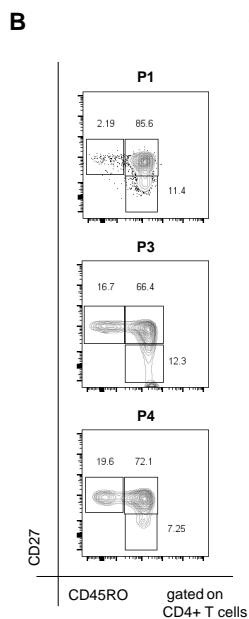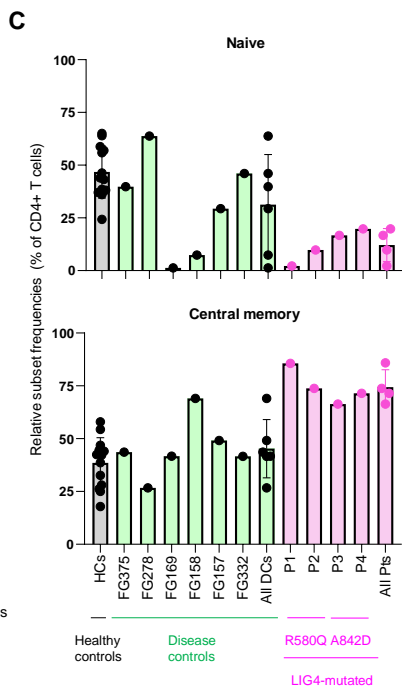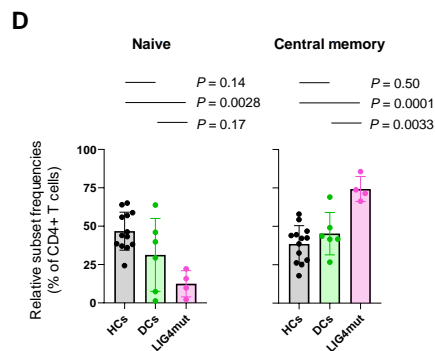

Supplement: Supplemental Figure E6 [file NIHMS1915164-supplement-Supplemental_Figure_E6.pdf]
